# Breast Cancer Cryoablation in the Multidisciplinary Setting: Practical Guidelines for Patients and Physicians

**DOI:** 10.3390/life13081756

**Published:** 2023-08-16

**Authors:** Dennis Holmes, Geeta Iyengar

**Affiliations:** 1 Adventist Health Glendale, 1505 Wilson Terrace, Suite 370, Glendale, CA 91206, USA; 2 Medical Imaging Center of Southern California, 8727 Beverly Blvd., Beverly Hills, CA 90048, USA

**Keywords:** cryoablation, breast cancer, ablation, alternative, cryosurgery, multidisciplinary, guideline

## Abstract

Breast cancer cryoablation has emerged as a minimally invasive alternative to lumpectomy for treating early-stage breast cancer. However, no consensus exists on what should be considered the standard of care for the multidisciplinary management of patients treated with breast cancer cryoablation. In lieu of national guidelines, this review of the literature provides a multidisciplinary framework and an evidence-based discussion of the integration of “standard of care practices” in the comprehensive management of breast cancer cryoablation patients.

## 1. Introduction

Over the last 10 years, breast cancer cryoablation has emerged as a minimally invasive alternative to lumpectomy for the management of early-stage breast cancer. Despite the rising popularity of breast cancer cryoablation, there remains no consensus on what should be considered the standard of care for breast cancer patients treated with cryoablation, which leaves many clinicians at a loss about how to manage cryoablation patients in a multidisciplinary context. In lieu of a national consensus, presented herein is an evidence-based practical guideline for the multidisciplinary management of breast cancer cryoablation patients.

Breast cancer cryoablation utilizes extremely cold temperatures to kill cancer cells along with a surrounding margin of normal breast tissue, comparable to what would be achieved with lumpectomy. Several potential advantages make cryoablation an appealing alternative to lumpectomy. In the context of patient-centered care, cryoablation expands breast cancer treatment options for patients seeking to avoid conventional surgery due to philosophical motivations, health concerns, or other reasons. As a percutaneous, office-based procedure performed under local anesthesia, cryoablation minimizes or eliminates the potential morbidity of breast cancer surgery (e.g., symptomatic seroma, hematoma, infection) and general anesthesia (e.g., nausea, vomiting, and exacerbation of cardiopulmonary conditions). Compared to breast cancer surgery, cryoablation has a minimal impact on quality of life and permits early resumption of work and daily activities. A breast cryoablation procedure video can be viewed at https://youtu.be/vSLbVy8BVLA (accessed on 12 August 2023).

Unlike lumpectomy, cryoablation does not involve the excision of breast tissue and thereby eliminates many of the adverse cosmetic effects of tumor excision. As such, cryoablation is the ultimate esthetic solution for breast cancer, reducing the need for pre-emptive or corrective surgical procedures aimed at maintaining or restoring breast volume, contour, and symmetry.

There are currently no published randomized controlled trials directly comparing the effectiveness or safety of cryoablation to lumpectomy. However, an ongoing single-center, prospective, randomized controlled trial at Washington University in St. Louis, Missouri aims to compare cryoablation to lumpectomy among 256 participants [1]. Study inclusion criteria are primarily limited to women aged 50 and older with stage I (≤2 cm), estrogen receptor positive (ER+) and/or progesterone receptor positive (PR+), HER2/neu negative, Luminal A subtype, grade I or II invasive ductal carcinoma.

In the absence of completed randomized trials, patients and providers can be encouraged by the results of the ICE3 Trial, a prospective, non-randomized, single arm, multicenter, U.S.-based study that demonstrated a 2.06% 3-year local recurrence rate among women treated with cryoablation in lieu of lumpectomy [2]. The main inclusion criteria were age 60 and older, stage I (≤1.5 cm), ER+ and/or PR+, HER2/neu-negative, and invasive ductal cancer. Seventy-five percent of ICE3 trial participants received endocrine therapy and only 14% received radiation therapy. Despite the infrequent use of adjuvant radiation, the low local recurrence rate observed in the ICE3 Trial is comparable to local recurrence rates reported by landmark clinical trials of lumpectomy and radiation.

The efficacy of breast cancer cryoablation is also supported by an ongoing prospective, single center clinical trial conducted at the Kameda Medical Center (Kamogawa, Chiba, Japan). Trial inclusion criteria are limited to women with stage I (≤1.5 cm), clinically node-negative, grade 1 or 2, ER+ and/or PR+, HER2/neu negative, invasive ductal carcinoma. All patients received cryoablation followed by sentinel node biopsy, anti-estrogen therapy, and whole breast radiation therapy. Although results of the Kameda study have yet to be published in manuscript form, an oral presentation at the 2019 American Society of Breast Surgeons annual meeting reported a 0.98% local recurrence rate among 304 patients of mean age 57 (31–83 years) with a median follow-up of 6 years, which demonstrates the ability of cryoablation combined with adjuvant therapy to achieve long-term local control in a suitable patient population.

Although breast cancer cryoablation technology is optimized for the treatment of ultrasound visible stage I breast cancers, technique modifications can permit cryoablation of Stage 0, II, III, and IV breast cancer when patient consent or healthcare resources restrict treatment options, as discussed in “Breast Cancer Care During a Pandemic: An Opportune Time for Cryoablation” [3]. Table 1 identifies several clinical scenarios for which cryoablation might be considered in the context of shared decision-making between a patient and her/his healthcare provider(s). In the table, “definitive therapy” refers to the use of cryoablation to treat breast cancer without a plan for subsequent surgical removal, provided that the targeted tumor can be cryoablated completely. “Stopgap therapy” refers to the use of cryoablation as a temporary solution prior to anticipated surgical resection.

## 2. The Pre-Treatment Assessment

The last two decades have seen steady improvement in breast cancer survival rates and a significant reduction in treatment-related morbidity. Although a major source of this progress has been early detection coupled with timely treatment, other key reasons for these improvements include the appropriate escalation or de-escalation of cancer therapy based on tumor biology, the use of targeted anticancer medications based on tumor biology, improvements in surgical techniques, and the adoption of diagnostic and treatment protocols that emphasize evidence-based practices while minimizing interventions with marginal clinical benefit. Despite these advancements, a common characteristic among patients seeking breast cancer cryoablation is a desire to avoid certain evidence-based practices for managing their breast cancer. Ultimately, the long-term success of cryoablation depends less on the cryoablation procedure itself, but more on the measures that are taken to assess the extent of disease and minimize the risk of local, regional, and systemic recurrence. Therefore, even in the context of patient-centered care, proper informed consent requires that patients understand the role that key quality metrics play in optimizing appropriate patient selection, proper treatment sequencing, and long-term cancer outcomes.

Care Coordination. The complexity of breast cancer care benefits from care coordination between multiple cancer specialists, each of whom requires specific pre-treatment and post-treatment assessments to determine the best course of care. A major hazard faced by patients seeking non-standard breast cancer treatment options such as cryoablation is that they are often left to navigate themselves along a complex cancer journey without the steadfast guidance and support of an experienced breast surgeon, surgical oncologist, medical oncologist, or team of cancer specialists. As a result, these patients often have an incomplete understanding of the extent of their disease and risk of recurrence; a poor understanding of the benefits and risks of various treatment options; a lack of appropriate, coordinated specialist referrals (even if specialist recommendations are ultimately declined); inappropriate or inadequate use of cancer staging studies; underuse of genomic and genetic testing; and insufficient use of lymph node staging, systemic therapy and adjuvant radiotherapy. Although patients seeking standard treatments might also experience these challenges, for cryoablation patients, the likelihood of experiencing these hazards is heavily influenced by the specialist that performs the cryoablation procedure and whether that individual is directly or indirectly accountable for other aspects of the patient’s overall cancer care.

In the U.S., surgeons and radiologists perform the vast majority of breast cancer cryoablation procedures. The major advantage of surgeon-performed cryoablation is that breast surgeons and surgical oncologists customarily assume responsibility for either administering or coordinating all aspects of a patient’s initial phase of breast cancer care. As accountable providers, breast surgeons and surgical oncologists are often key decision-makers regarding the proper sequence of cancer care (surgery first vs. upfront or neoadjuvant systemic therapy), the need for genetic and genomic testing, the appropriate use of breast cancer staging, the appropriate management of the lymph nodes (observation vs. surgery), and the need for and order of priority of referral to medical oncology, radiation oncology, and other ancillary services, with the overall goal of minimizing the risk of local, regional and systemic recurrence. Surgeons routinely counsel patients about and have the capacity to perform lymph node surgery, when indicated, as well as other surgical alternatives to cryoablation (e.g., lumpectomy, oncoplastic surgery, and mastectomy +/− reconstruction). Surgeons may also initiate anti-estrogen therapy as stopgap or adjuvant therapy. 

On the other hand, radiologists primarily focus on the technical aspects of breast cancer ablation, but relatively few assume the role of the accountable provider to the degree that is required for a patient facing a potentially life-threatening cancer diagnosis. Consequently, it is advisable for a radiology cryoablation provider to partner with a surgeon and/or medical oncologist who will accept responsibility for managing or coordinating the patient’s multidisciplinary cancer care. When such collaboration is absent, breast cryoablation patients are at greater risk of receiving incomplete or substandard multidisciplinary cancer care. This is not to minimize the importance of radiologists to a successful cryoablation procedure, as they are crucial for detecting breast cancer, evaluating the extent of breast and axillary disease, and performing localization procedures that facilitate cryoablation of non-ultrasound visible cancer. 

A high level of competency is also required of surgeons who perform cryoablation. Although many surgeons possess basic ultrasound skills, breast cancer cryoablation requires advanced skills in ultrasound interpretation and performance of ultrasound-guided procedures. Therefore, surgeons performing breast cancer cryoablation should receive and maintain formal ultrasound certification by the American Society of Breast Surgeons, American College of Radiology, or similar professional societies. In the absence of ultrasound certification, patient outcomes might be compromised.

Regardless of the provider, the best long-term results following breast cancer cryoablation are derived when both the provider and the patient have a comprehensive understanding of the key quality metrics regarding the diagnostic work-up, pre-treatment planning, and follow-up of patients seeking or previously treated with breast cryoablation. These key quality metrics are summarized in Table 2 and elaborated upon in the following discussion.

**Figure 1:**
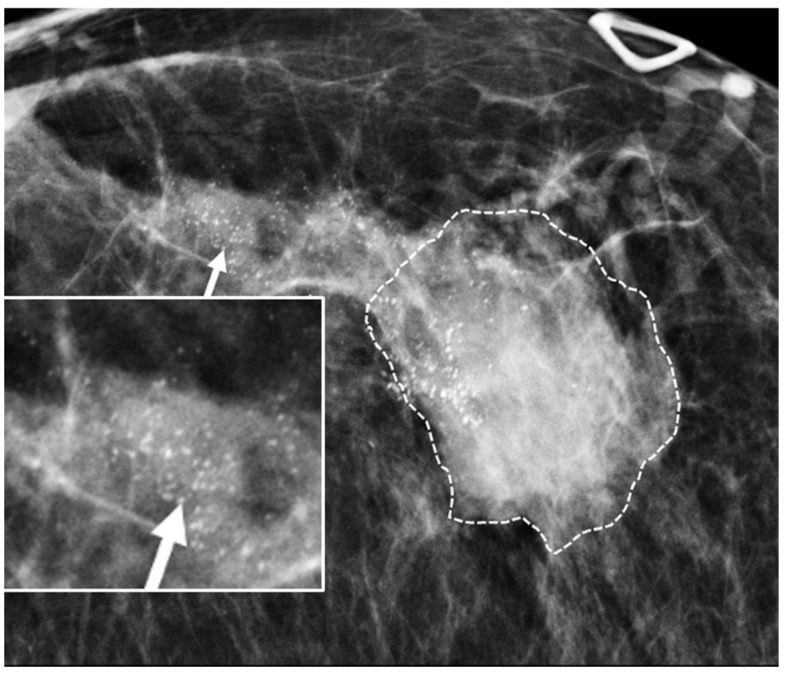
Mammogram showing density corresponding to a palpable mass (see dashed lines) as well as suspicious microcalcifications (arrows) and inset image showing magnified view of white punctate calcifications extending beyond the palpable mass.

Digital Mammography. Breast cancer staging should include bilateral digital diagnostic mammograms (preferably 3D mammograms or tomosynthesis) to evaluate the extent of disease. Although cryoablation is generally performed under ultrasound or CT-scan guidance, high-quality mammography might reveal additional areas of cancer in the same region of the breast (multifocal cancer) or in a different region of the breast (multicentric cancer) that might impact patient selection or alter the cryoablation treatment plan. Multicentric and multifocal breast cancer is generally regarded as a contraindication to cryoablation. Pre-treatment assessment of the mammograms should seek the detection of spiculations (radial tumor extensions) and/or microcalcifications that might reach beyond the main tumor mass and require incorporation in the cryoablation treatment zone (Figure 1). Since mammography is the only imaging study that reliably shows microcalcifications, mammography plays a particularly important role in determining disease extent when suspicious microcalcifications are part of the disease process. Consequently, patients who are generally opposed to obtaining screening mammograms should be encouraged to have at least one set of pre-treatment diagnostic mammograms to exclude the presence of suspicious microcalcifications that could impact patient selection and/or treatment planning.

**Figure 2:**
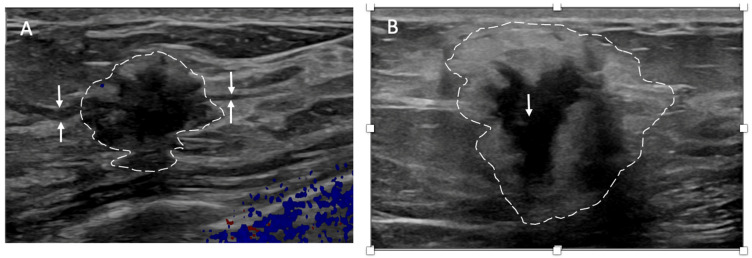
(**A**) shows dark, dominant, irregular mass encircled by hash marks with intraductal tumor extensions (dark bands bracketed by paired arrows) extending from left and right sides of dominant mass. (**B**) shows dark, irregular dominant mass (arrow) surrounded by peri-tumoral edema outlined by hash marks.

Ultrasound of the breast. Office-based cryoablation is optimized for treatment of ultrasound-visible breast cancers. Consequently, breast ultrasound should be performed of all suspicious imaging abnormalities to assess eligibility for ultrasound-guided cryoablation. Ultrasound permits assessment of the cancer’s proximity to the overlying skin and underlying chest wall and also enables detection of changes in adjacent tissue architecture (e.g., edema, tissue distortion, extension of tumor into adjacent ducts) and could indicate the presence of more extensive disease that would need to be incorporated into the treatment plan (Figure 2). Proximity (<5 mm) of a cancer to the skin is generally regarded as a contraindication to cryoablation, but a safe skin distance can sometimes be created using hydrodissection or injection of saline between the tumor and overlying dermis. 

**Figure 3:**
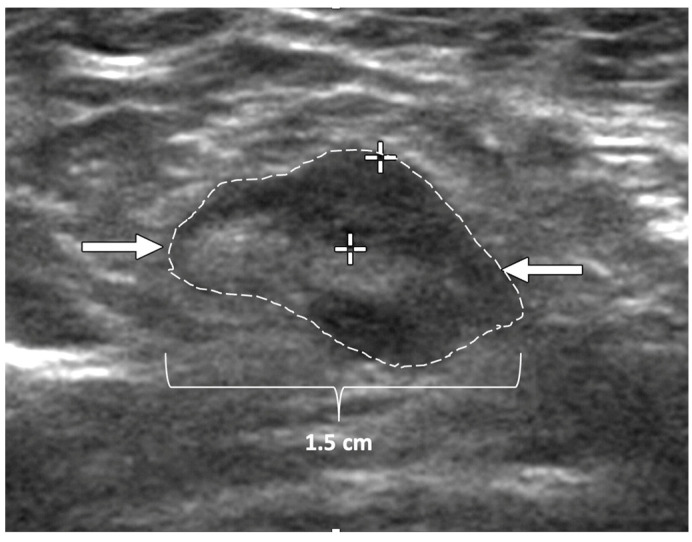
Hash marks outline abnormal appearing axillary lymph node measuring 1.5 cm in maximal diameter. Paired “+” marks indicate the span of a 0.4 cm area of focal cortical thickening that is suspicious for a metastatic deposit within the lymph node.

Ultrasound of the axilla. Axillary ultrasound permits the detection of abnormal appearing lymph nodes that might not be detected on physical examination, mammography, or breast MRI. Axillary ultrasound is the most sensitive imaging study for the detection of subtle changes in the shape or thickness of a lymph node cortex that might indicate the presence of lymph node metastasis (Figure 3). Detection of suspicious lymph nodes determines clinical cancer stage and requires a specific plan to manage the possibility of lymph node metastasis.

**Figure 4:**
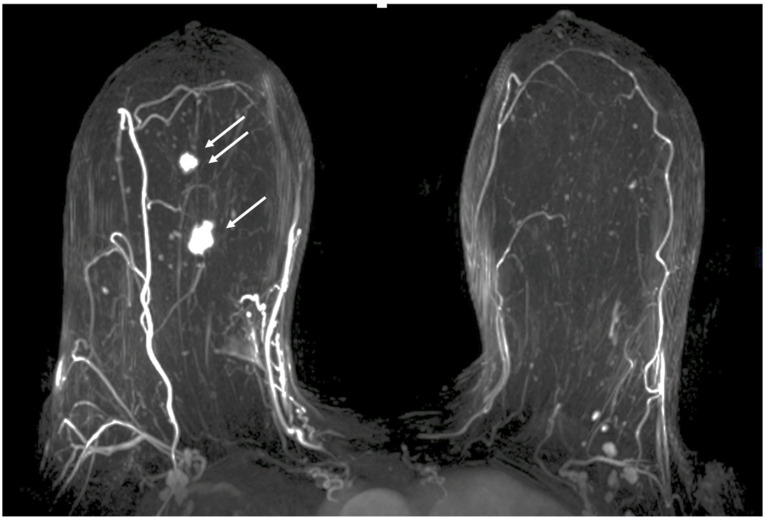
Contrast-enhanced breast MRI image showing multifocal disease indicated by mass-like enhancement of dominant mass (indicated by single arrow and encircled with hash marks) as well as mass-like enhancement of a second focus of disease (indicated by double arrows and encircled with hash marks). The second focus was mammographically-occult.

Contrast-Enhanced Breast MRI. Contrast enhanced breast MRI improves assessment of disease extent, including detection of spiculations, multifocal, multicentric, or contralateral disease, which might impact eligibility for cryoablation or alter the overall treatment plan (Figure 4). Contrast-enhanced breast MRI has the added value of permitting 3-D image reconstruction to permit viewing of the tumor from various angles for a more comprehensive assessment of tumor shape and size. Breast MRI for cancer evaluation requires the use of an intravenous contrast containing gadolinium that can be safely administered with minimal short-term and long-term risks. However, patients unwilling or unable to receive gadolinium might be able to access dedicated breast-only diffusion weighted MRI, which has limited availability in the U.S.

Thermography. Many cryoablation patients have undergone thermography as a supplement to or substitute for mammography. Thermography aims to detect cancer using an infrared camera to capture from the surface of the breast heat that is emitted by an underlying tumor. Some cryoablation candidates express a preference for thermography to avoid mammogram-related radiation and breast compression. Despite the apparent advantages of thermography, multiple studies comparing thermography to mammography or thermography to ultrasound have found thermography to be inferior to either modality for detection of breast cancer [4,5]. Therefore, thermography also should not be used to exclude a suspicious abnormality detected by mammography, ultrasound, or breast MRI. Furthermore, any suspected cancer detected by thermography should be evaluated with mammography and ultrasound to characterize the extent of disease, to guide a diagnostic needle biopsy and to assess its suitability for ultrasound-guided cryoablation. 

Breast needle biopsy. Core needle or fine needle biopsy of the breast tumor should be performed under mammogram, ultrasound, or MRI-guidance to establish the diagnosis of cancer and to confirm or exclude the presence of multifocal or multicentric disease, if suspected. However, some patients seeking cryoablation are opposed to having a diagnostic core needle biopsy of the tumor performed due to concerns that the needle biopsy itself might cause cancer spread. Although many of these patients will ultimately agree to undergoing needle biopsy after being reassured that the risk of needle biopsy-induced metastasis is rare, others can only be convinced to undergo a needle biopsy as part of the cryoablation procedure when tumor cells potentially dislodged by the needle biopsy could be immediately killed by cryoablation. Unfortunately, this same day diagnostic needle biopsy-cryoablation procedure renders the patient ineligible for pre-cryoablation anticancer medications, if appropriate, that could be beneficial to her overall survival. However, the very decision to forego a needle biopsy precludes the use of pre-cryoablation anticancer medications. 

Although there is no evidence linking the needle biopsy procedure itself to a reduction in breast cancer survival, there is clear evidence that avoidance of a diagnostic needle biopsy contributes to cancer growth and spread which reduces overall survival. According to Rutter et al., a 3 month or longer delay between abnormal imaging and a positive needle biopsy is associated with an increased incidence of late-stage disease and reduced long-term survival [6]. Thus, the individuals who are most averse to a needle biopsy are at the greatest risk of delayed diagnosis and delayed treatment explicitly due to their decision to avoid a needle biopsy.

**Figure 5:**
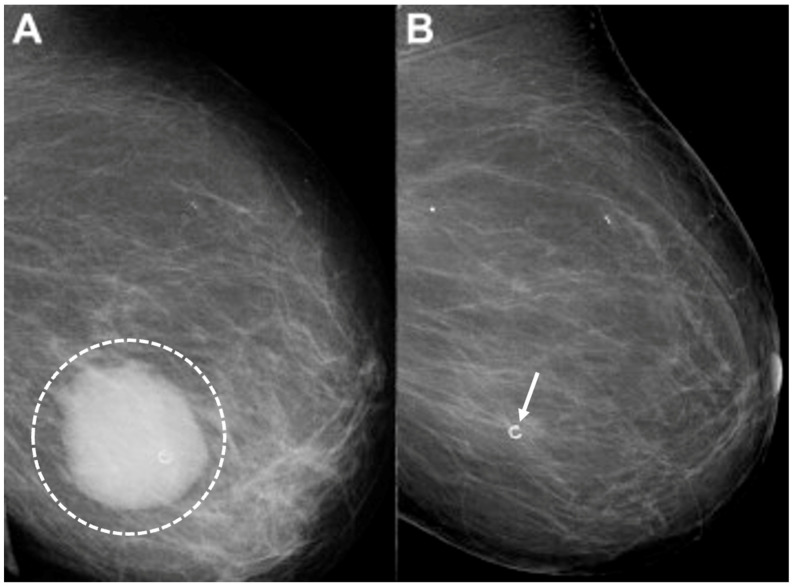
Mammogram performed before (**A**) pre-operative chemotherapy showing large, white, ultrasound visible cancer encircled by hash marks. After completing pre-operative chemotherapy, a repeat mammogram (**B**) of the same breast showed complete disappearance of the original cancer leaving only a circular metal biopsy site marker (arrow) to indicate the location of the original cancer.

Placement of biopsy site marker(s). Regardless of cancer size, insertion of a biopsy site marker (also called a “clip” or “chip”) at the time of mammogram-, ultrasound-, or MRI-guided needle biopsy is recommended to document the site of the cancer. In situations where pre-cryoablation chemotherapy and/or anti-HER2/neu therapy is planned, insertion of a biopsy site marker prior to initiation of anticancer medications documents the location of the cancer in the event drug therapy-induced shrinkage of the cancer makes it difficult to detect by ultrasound (Figure 5).

**Figure 6:**
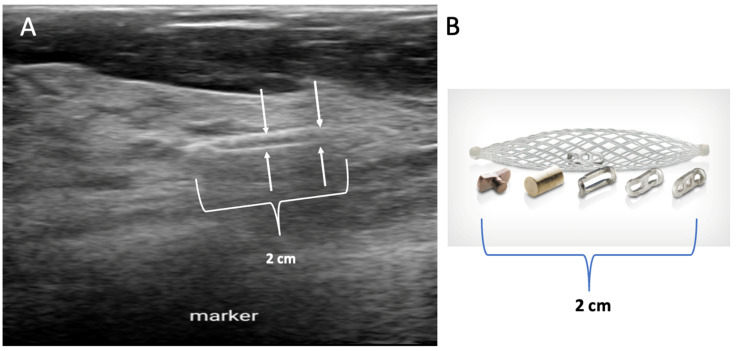
Ultrasound image (**A**) showing an example of a 2 cm long, basket-like, tubular, ultrasound-visible biopsy site marker (bracketed by paired arrows) that documents the location of ductal carcinoma in situ, which itself was not ultrasound visible. Image (**B**) shows an enlarged view of an actual basket-like marker and the shapes of various radio-opaque markers they may contain.

Biopsy site markers come in many shapes and sizes, but most measure less than 10 mm in length. In breast radiology, the most commonly used biopsy site markers are metallic (stainless steel, titanium, or nickel) devices that are optimized for detection by mammography, but are poorly visible by ultrasound. However, several biopsy site markers have been optimized for ultrasound detection by encasement of the metallic component in a larger, absorbable collagen or hydrogel sleeve (Figure 6). For cancers that are already visible by ultrasound, the type of marker is unimportant as the tumor itself provides an obvious target for ultrasound-guided cryoablation. However, when the breast cancer is very small or invisible to ultrasound, insertion of an ultrasound-visible marker at the time of the initial needle biopsy or subsequently will facilitate ultrasound-guided cryoablation of ultrasound-occult cancer.

**Figure 7:**
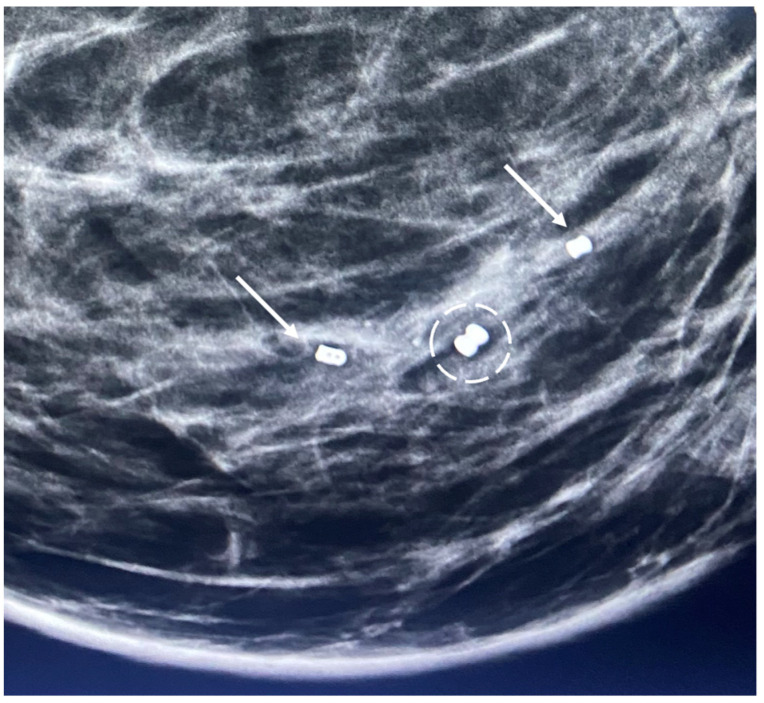
Mammogram showing the metal components (indicated arrows) of two ultrasound-visible biopsy site markers on opposite sides of the original metal biopsy site marker (encircled by hash marks) that was placed at the time of the diagnostic needle biopsy. The original biopsy site marker was not an ultrasound visible.

Generally, a single biopsy site marker is sufficient for marking the site of most breast cancers. However, patients with a wide span of disease may benefit from placement of two or more ultrasound-visible markers to outline or bracket the borders of the cancer to improve targeting of the cryoablation treatment (Figure 7).

Ductal carcinoma in situ (DCIS) poses a unique challenge for cryoablation. DCIS is most often detected by mammography based on the presence of associated radio-opaque microcalcifications that are difficult to detect by ultrasound. The lack of ultrasound visibility accounts for why DCIS has been excluded from nearly all previous clinical trials of ultrasound-guided cryoablation. However, the ability to insert an ultrasound-visible biopsy site marker at the site of DCIS expands the possibility of performing cryoablation for DCIS. The role of cryoablation in the management of DCIS is currently being examined in a prospective clinical trial assessing the role of cryoablation as a minimally invasive alternative to surgery for preventing the progression of DCIS to invasive breast cancer [7]. The study is open to women aged 18 and older with unifocal DCIS measuring ≤2 cm by mammography and optional breast MRI. All subjects with non-ultrasound visible DCIS undergo the insertion of an ultrasound-visible marker at the site of DCIS. Marker placement is followed by ultrasound-guided cryoablation without subsequent excision. Six months after cryoablation, all subjects undergo a mammography-guided needle biopsy to confirm complete DCIS ablation followed by annual mammography (if biopsy negative) or excision (if biopsy positive). The primary endpoint is complete ablation of malignancy assessed 6 months post-ablation. The secondary endpoint is the 5-year rate of invasive breast cancer recurrence. A second study is a prospective clinical trial of women with unifocal DCIS measuring ≤1.5 cm by mammogram, ultrasound, and breast MRI [8]. All subjects will undergo ultrasound-guided cryoablation of DCIS following placement of an ultrasound-visible marker, if needed. Following cryoablation, all subjects undergo surgical excision of the cryoablation site to determine the rate of complete ablation of malignancy in the surgical resection specimen. In both studies, the use of adjuvant endocrine therapy and radiation therapy is left to the discretion of the physician and patient.

Lymph Node Needle Biopsy. Ultrasound-guided core needle biopsy or fine needle aspiration of a suspicious axillary node should be performed to document metastasis to the axillary nodes. Insertion of an ultrasound-visible biopsy site marker at the time of the initial biopsy, or subsequently, will facilitate ultrasound-guided cryoablation or ultrasound-guided excision of the lymph node should it become less detectable after the needle biopsy or after pre-cryoablation anticancer medications.

Determination of cancer biomarkers (prognostic markers or receptors). Historically, cancer biomarkers were primarily measured by pathologists in the breast cancer specimen removed by surgery. However, since the timing of surgery (or cryoablation) is potentially influenced by the level of cancer biomarker expression, it is now standard practice for pathologists to report cancer biomarkers levels measured in the diagnostic needle biopsy specimens. Following this principle, estrogen receptor (ER), progesterone receptor (PR), HER2/neu receptor, and preferably ki-67 should be tested in invasive breast cancer and reviewed by the cryoablation provider prior to initiating treatment. Similarly, ER and ideally PR expression should be assessed in ductal carcinoma in situ prior to treatment.

Measurement of cancer biomarkers in the needle biopsy specimen prior to surgery or cryoablation allows the provider and patient to determine if the tumor is ER+ and/or PR+ (i.e., hormone receptor positive), HER2/neu positive, or triple negative (i.e., ER-, PR-, and HER2/neu negative) which has implications for how the cancer is treated, particularly for invasive breast cancer. For example, hormone receptor positive, HER2/neu negative invasive breast cancer is generally managed with upfront surgery (or cryoablation) unless the tumor biology and the clinical scenario suggest a clear benefit to receiving upfront chemotherapy. On the other hand, triple negative invasive breast cancer and HER2/neu positive invasive breast cancer >2 cm are generally treated with upfront chemotherapy and/or anti-HER2/neu therapy with the potential for improved breast cancer survival compared to post-operative (or post-cryoablation) treatment. Although many cryoablation patients may be disinclined to receive chemotherapy, anti-estrogen therapy, and/or anti-HER2/neu therapy either before or after cryoablation, awareness of the potential survival benefits of pre-cryoablation anticancer medications might open their minds to receiving upfront anticancer medications, especially if this treatment approach does not preclude them from later receiving cryoablation.

**Figure 8:**
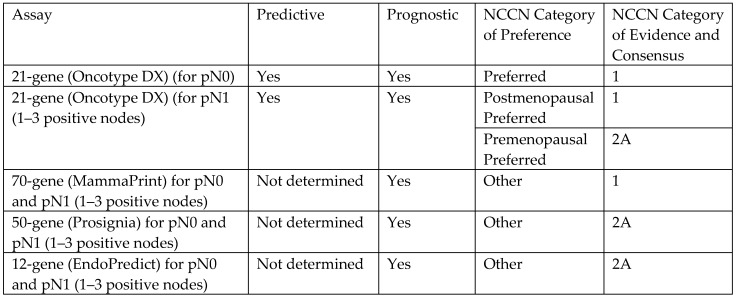
Table showing commercially available genomic assays for invasive breast cancer as indicated by the National Comprehensive Cancer Network guideline.

Genomic testing. Genomic testing (e.g., OncotypeDX, MammaPrint, Prosignia, EndoPredict) is an important predictive and/or prognostic strategy for estimating the 5-year or 10-year risk of invasive breast cancer recurrence within the body and the potential benefit of adjuvant chemotherapy in a patient who is expected to be treated with anti-estrogen therapy (Figure 8) [9]. Although genomic testing is most commonly performed on the breast surgical resection tissue sample, genomic testing assays may be run on breast needle biopsy tissue samples obtained prior to cryoablation or surgery. In addition to quantifying the potential benefit of chemotherapy, clarifying the overall risk of recurrence can also influence the decision-making of a cryoablation candidate who is also considering skipping anti-estrogen therapy. For example, a high-risk genomic recurrence score might motivate a patient to initiate and remain compliant with anti-estrogen therapy, even if they declined chemotherapy. Conversely, a low-risk recurrence score might reduce the anxiety of a patient who has elected to decline or limit the duration of anti-estrogen therapy. However, since all patients with triple negative or HER2/neu positive invasive breast cancer would receive a high-risk recurrence score, genomic testing has no role in estimating the benefit of systemic therapy in these patients.

Another potential role for genomic testing for hormone receptor positive, HER2/neu negative invasive breast cancers, is risk stratification for a patient who is considering declining radiation therapy or sentinel node biopsy. Despite being off-label uses of genomic testing, the rationale for utilizing genomic testing in this manner is supported by evidence showing a direct correlation between a high genomic recurrence score and an elevated risk of local recurrence or lymph node involvement [10]. 

For women with pure DCIS, there are currently two commercially available genomic tests (DCISionRT and OncotypeDX DCIS) for estimating the 10-year risk of invasive or DCIS recurrence within the breast after lumpectomy, with and without the addition of radiation therapy [11,12]. Since the predictive and prognostic values of DCIS genomic tests are based on DCIS biology, not the manner of treatment, it is reasonable to utilize DCIS genomic testing to estimate the potential benefit of radiation therapy and/or anti-estrogen therapy following cryoablation of DCIS.

Staging for Distant Metastasis. It is reasonable to assume that the principles guiding cancer staging for cryoablation patients would mirror the staging recommendations outlined in the most recent version of the NCCN guideline. For example, the current version of the NCCN guideline recommends routine whole-body scans only for patients with stage III or IV breast cancer for detection of metastasis in the lung, liver, or other internal organs [9]. Routine staging for stage III and IV breast cancer may consist of computed tomography (CT) scans of the chest and abdomen, with or without pelvic scans or 18F-fluorodeoxyglucose positron emission tomography (FDG-PET/CT) scans. Although many bone metastases may be detected by FDG-PET/CT, dedicated staging for bone metastases may be accomplished with a nuclear medicine bone scan or a sodium fluoride PET/CT scan.

For all other patients, the national guideline recommends sign or symptom-based screening. For example, a chest CT with and without contrast is appropriate for symptoms of cough, shortness of breath, etc.; abdominal +/− pelvic CT with contrast or abdominal MRI with contrast may be obtained to evaluate abnormal liver laboratory studies or symptoms of nausea or abdominal pain; spine MRI with contrast may be used to evaluate back pain; bone scan or sodium fluoride PET/CT may be obtained for bone pain; and brain MRI may be used to assess headache, dizziness, or other neurological symptoms. Chest X-rays are not recommended for routine breast cancer staging. Routine complete blood count, complete metabolic panel, liver function tests, and other serological studies are recommended when systemic therapy is being considered.

Genetic counseling and testing. Only 5–10% of breast cancers originate from hereditary breast cancer mutations. Nonetheless, the potential implications of a positive genetic test call for vigilance in identifying all pathogenic mutation carriers. Based on the current NCCN guideline, genetic counseling and testing should be considered for a variety of clinical scenarios, including a personal diagnosis of triple negative invasive breast cancer; a personal or close family history of breast cancer ≤ 50 years of age; a personal history of multiple primary breast cancers; or a personal or family history of either ovarian cancer, pancreatic cancer, or high-risk/metastatic prostate cancer [13].

A positive genetic test for a hereditary breast cancer does not eliminate the possibility of cryoablation. However, the informed consent discussion for mutation carriers should acknowledge the relatively higher risk of local recurrence when treated with breast preserving procedures [14]. Nonetheless, there should be no anticipated adverse impact on short-term and long-term survival when cryoablation is combined with appropriate anti-cancer medications and adjuvant radiation therapy. A positive genetic test result should also trigger referral of the cryoablation patient to a genetic counselor for management of other at-risk organs (e.g., ovaries) and for genetic counseling and testing of family members.

Timing of treatment. Patients seeking alternative treatments often experience significant delays of care as they consider their treatment options. Unfortunately, a growing body of evidence associates delayed initiation and delayed completion of cancer care with reduced cancer survival [15,16,17]. The survival implications of delayed breast cancer treatment were brought to light by recent analyses of the National Cancer Database Registry and the Surveillance, Epidemiology, and End Results-Medicare Program Database, which revealed a statistically significant 9–10% (*p* < 0.001) relative reduction in 10-year and 15-year overall survival for each additional 30-day delay when the time interval between a positive diagnostic needle biopsy and surgery (“time to surgery”) exceeded 60 days [18]. In this population-based study, the overall survival reduction was found to disproportionately affect women with stage I and II breast cancer (hazard ratio: 1.09–1.16, *p* < 0.001), which is the largest cohort of patients diagnosed with breast cancer annually and the group for which a survival reduction should be least tolerated given their otherwise excellent survival prospects.

With respect to breast cancer cryoablation, these time to treatment findings lead to two key recommendations. Firstly, patients should ideally undergo cryoablation within 60 days of their date of diagnosis to avoid compromising their overall survival. Secondly, patients who remain undecided about standard therapy or whose access to or utilization of standard therapy is delayed, may consider undergoing cryoablation as a stopgap measure to prevent ongoing tumor growth and spread.

## 3. Treatment Approaches

**Figure 9:**
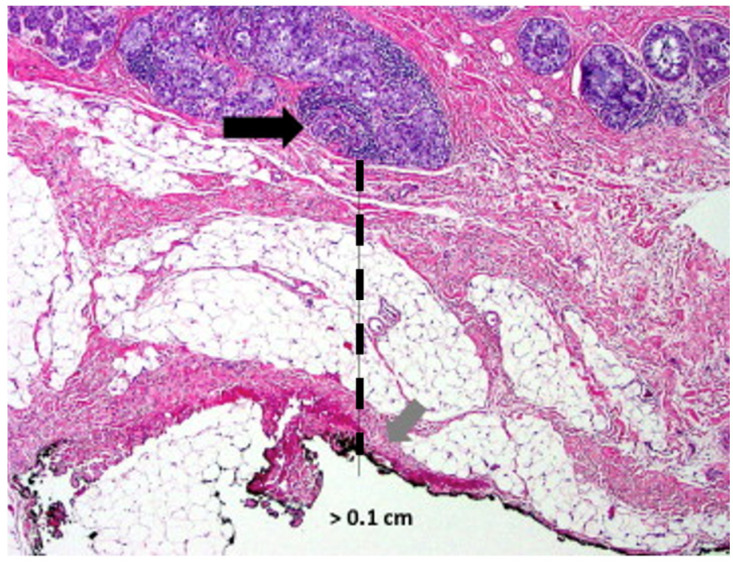
Microscopic image of a lumpectomy specimen showing clusters of cancer cells (arrow) approximately 1 mm (hash line) from the nearest surgical margin.

Successful breast cancer cryoablation requires a comprehensive understanding of the 3-dimensional extent of the cancer, including the presence and extent of satellite tumors, suspicious calcifications, or changes in tissue architecture that suggest the presence of additional microscopic disease. Once the extent of disease has been determined, successful cryoablation also requires conformation of one or more cryoablation zone(s) of necrosis to encompass the entire 3-dimensional volume of disease as well as a surrounding ablation margin of normal breast tissue. In contrast with surgery where a 1–2 mm microscopic surgical margin is acceptable, the inability to perform microscopic margin assessment with cryoablation necessitates a wider ablation margin surrounding the cancer to minimize the risk of leaving untreated residual disease in the perimeter of the main cancer mass (Figure 9). Furthermore, whereas a 5–10 mm ablation margin might be appropriate for a cancer with relatively distinct margins, wider cryoablation margins (10–20 mm) might be more desirable for a cancer that has indistinct margins or for situations when the patient intends to decline radiation therapy.

Single vs. multiple cryoablations. The most obvious physical effect of cryoablation is the generation of a palpable and ultrasound-visible iceball which corresponds to the cryoablation zone of injury at the center of which is the cryoablation zone of necrosis. Since the outer 0.5 cm edge of the cryoablation zone of injury is not cold enough to cause complete tissue necrosis, the iceball must attain a volume of 5 × 4 × 4 cm to achieve a 4 × 3 × 3 cm cryoablation zone of necrosis, which corresponds to a temperature of minus 20 °C or colder. 

**Figure 10:**
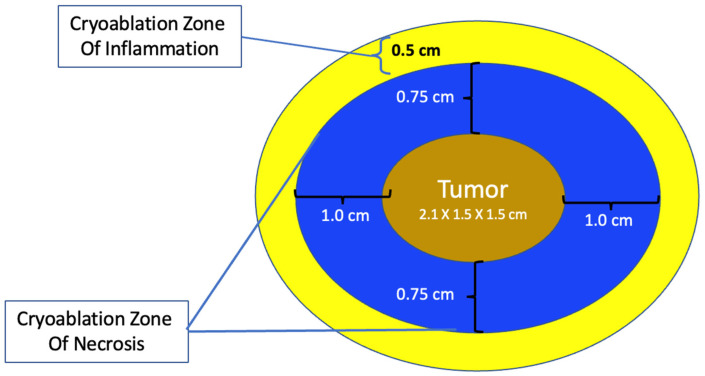
Schematic of cryoablation of a 2 × 1.5 × 1.5 cm tumor within a 4 × 3 × 3 cm cryoablation zone of necrosis and a surrounding 0.5 cm cryoablation zone of inflammation.

The total length of each cryoablation procedure depends on the volume of tissue to be ablated and the internal mechanics of the cryoprobe, which in turn determine the rate of flow of the liquid nitrogen or argon gas cryogen and the overall shape of the iceball. The typical cryoprobe used for breast cancer cryoablation generates an oval shaped iceball using two freeze cycles separated by a passive thaw cycle. For example, a 10-min freeze, 10-min thaw, 10-min freeze schedule generally achieves a cryoablation zone of necrosis measuring a maximum of 4 × 3 × 3 cm, sufficient for ablation of a single cancer ≤2 cm plus a surrounding “surgical margin” ≥ 5 mm. As a second example, for a 2 × 1.5 × 1.5 cm tumor oriented along the long axis of the cryoprobe, a 4 × 3 × 3 cm cryoablation zone of necrosis achieves a minimum gross surgical margin of 0.75 cm (Figure 10). Although a larger cryoablation zone of necrosis or wider ablation margins might be desired in certain cases, the fixed flow rate of the cryogen and competing warming effect of normal body temperature limits the potential to generate a larger cryoablation zone of necrosis using a single cryoprobe, even if freeze times are extended.

Modern technology for office-based breast tumor cryoablation is optimized for treatment of a single tumor ≤2 cm. However, clinical experience in cryoablation of larger cancers (e.g., liver cancers) supports the use of multiple, overlapping cryoablation zones of necrosis to encompass complete ablation of a large volume of tissue [19]. For example, argon gas-based cryoablation systems are well suited for the treatment of larger cancers since they permit the insertion of multiple adjacent cryoprobes within and surrounding a cancer to enable simultaneous ablation via all cryoprobes at once using only 2 or 3 freeze-thaw cycles. On the other hand, ablation of large cancers using a single cryoprobe system (either liquid nitrogen or argon gas-based) require multiple, consecutive, partially overlapping cryoablation treatments to achieve a larger volume of ablation than can be accomplished with a single cryoablation treatment.

Single cryoprobe systems have lower treatment costs, may be performed under local anesthesia, and are better suited for office-based treatment, but may require multiple consecutive cryoablation treatments, each approximately 30 min in length. On the other hand, multiple cryoablation systems permit faster treatment times, but have higher treatment costs (i.e., multiple cryoprobes), generally require general anesthesia or intravenous sedation, and are less well suited for office-based treatment.

**Figure 11:**
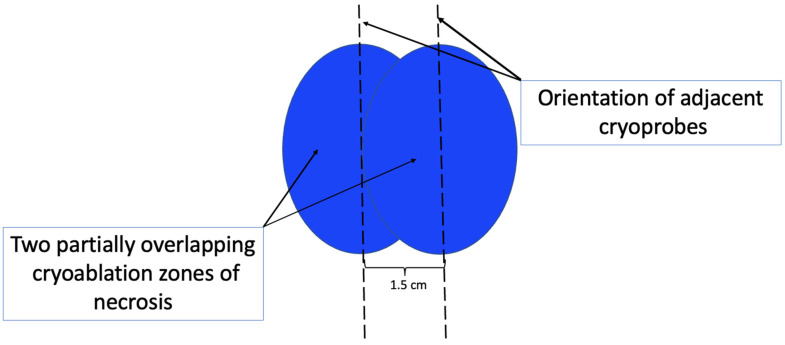
Schematic showing orientation (indicated by hash lines) and recommended spacing (1.5 cm) of adjacent cryoprobes for cancers requiring more than one cryoablation zone of necrosis to minimize gaps between adjacent cryoablation zones of necrosis. Cryoablation zones of inflammation are not shown.

**Figure 12:**
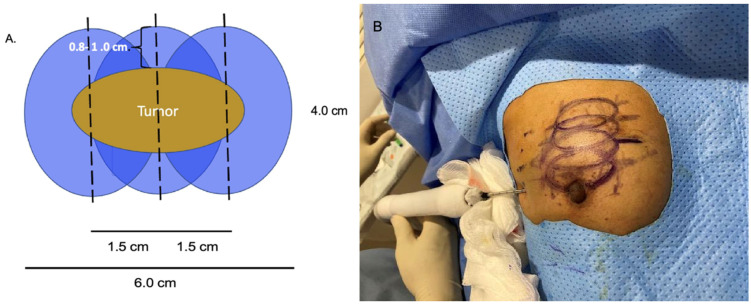
Schematic (**A**) of 4 × 2 × 2 cm tumor requiring 3 cryoablation zones of necrosis to achieve minimum ablation margins of 0.8–1.0 cm. Cryoablation zones of inflammation are not shown. Photo (**B**) shows the relative positions of 4 oval overlapping cryoablation zones of necrosis and 1.5 cm cryoprobe spacing for a multifocal cancer in the upper right breast.

**Figure 13:**
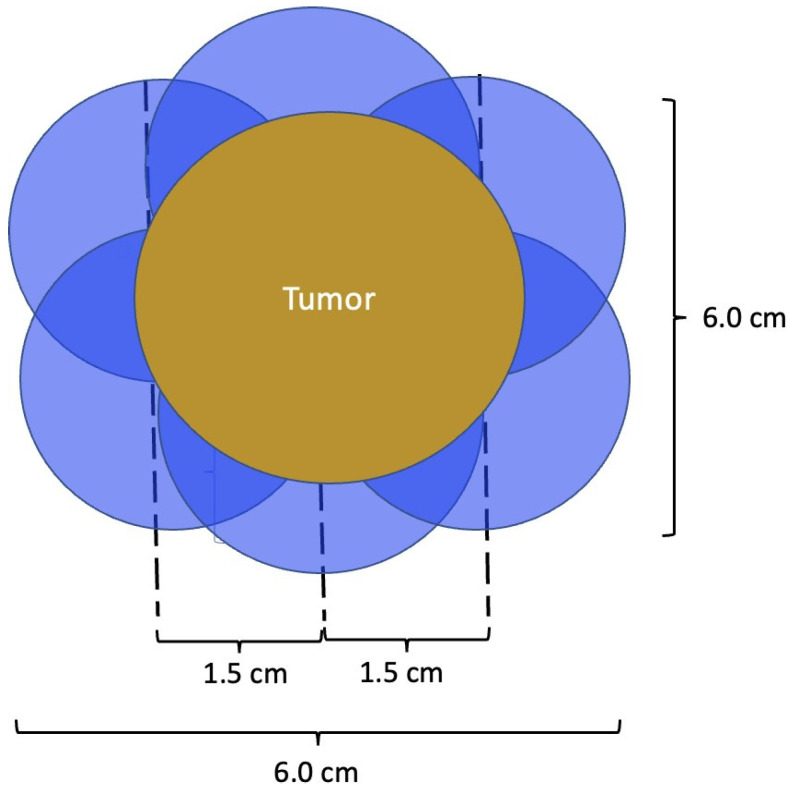
Schematic of a 4 × 4 × 4 cm tumor requiring a minimum of 6 cryoablation zones of necrosis to achieve minimum ablation margins of 1.0 cm. Cryoablation zones of inflammation are not shown.

At present, there is no official manufacturer’s guidance on the appropriate spacing between consecutive cryoprobe insertions when a single cryoprobe system is used for multiple cryoablation treatments. However, based on anticipated cryoablation zones of necrosis spanning 4 × 3 × 3 cm, clinical judgment supports spacing adjacent cryoprobe insertions 1.5 cm apart to minimize the risk of leaving non-ablated tissue between or within overlapping oval cryoablation zones of necrosis (Figure 11). Thus, using the “1.5 cm spacing principle” and 4 × 3 × 3 cm cryoablation zone of necrosis, cryoablation of a 4 × 2 × 2 cm cancer with a 1 cm peripheral ablation margin (i.e., a 6 × 4 × 4 cm volume) would require 4 (i.e., 6 cm divided by 1.5 cm) partially overlapping cryoablation zones of necrosis (Figure 12). By contrast, cryoablation of a 4 × 4 × 4 cm tumor with a 1 cm peripheral ablation margin (i.e., a 6 × 6 × 6 cm volume) would require at least 6 overlapping cryoablation zones of necrosis, which significantly increases the complexity, duration, and resource demands of the procedure (Figure 13).

Performing additional biopsies at time of cryoablation. Successful cryoablation means that there will be no residual cancer tissue available for pathological assessment after completion of the cryoablation procedure. Consequently, the physician performing the cryoablation procedure should ascertain whether additional biopsies of the cancer should be obtained immediately before beginning the cryoablation procedure. Potential reasons for performing additional biopsies include obtaining additional tissue for genomic testing, checking for the presence of residual disease at the tumor site after completing pre-cryoablation anticancer medications, or wider tissue sampling of a large area of DCIS to rule out hidden invasive carcinoma.

Cancer Localization. In most instances, ultrasound alone is sufficient for locating and guiding cryoablation of an ultrasound-visible cancer. However, when portions of the cancer are not fully ultrasound-visible (e.g., calcifications, spiculations, or MRI non-mass enhancement), then mammogram-guided or MRI-guided insertion of an ultrasound-visible biopsy site marker provides a surrogate target for locating and directing the cryoprobe for ultrasound-guided cryoablation.

There are three common scenarios related to cancer localization that must be considered prior to cryoablation, each of which can have a significant impact on the success of the cryoablation procedure. Firstly, the imaging features of the cancer are often completely removed or obscured by hematoma during the diagnostic needle biopsy procedure, making the original cancer difficult to detect in subsequent imaging. Therefore, when the patient presents for placement of an ultrasound-visible marker prior to cryoablation, the radiologist must utilize available tissue landmarks to place the marker within the span of the cancer based on its pre-biopsy dimensions, not simply based on the components that remain detected after the needle biopsy. Without this adjustment, the ultrasound visible marker might inadvertently be placed near the lateral edge of the original cancer, rather than at its center, which could result in off-centered placement of the cryoprobe, possibly resulting in incomplete cancer ablation and/or inadequate margins.

**Figure 14:**
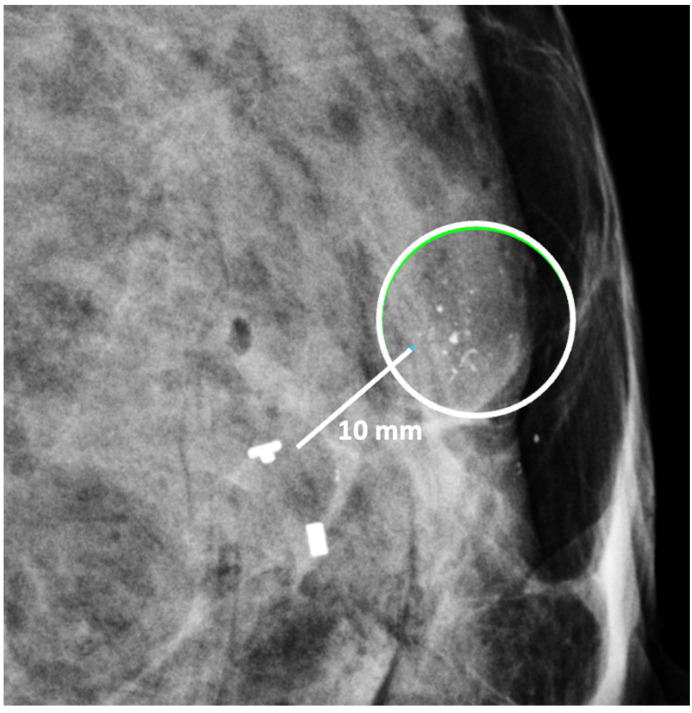
Mammogram showing clip migration of two biopsy site markers displaced ~10 mm from microcalcifications (encircle). The lower, bar-shaped marker was placed at the time of the original diagnostic needle biopsy. The upper, T-shaped ultrasound-visible marker was subsequently inserted for localization of the calcifications, but also migrated ~10 mm from the intended location.

The second important consideration is marker migration or displacement. When a biopsy site marker is inserted at the time of the needle biopsy procedure, the marker may sometimes migrate or move several millimeters or centimeters away from its intended location along the needle tract (Figure 14). Marker migration > 10 mm occurs in 20% of diagnostic needle biopsy procedures and is more likely to occur with markers placed under mammogram or MRI-guidance, where the marker may shift away from its intended site when the breast is released from compression [20]. Bleeding or hematoma resulting from the needle biopsy can also displace a marker from its original location. Regardless of the cause, if adjustments are not made for marker migration, there is a reasonable possibility that the cancer will be incompletely ablated or missed altogether.

Lymph Node Management. The decision to undergo cryoablation of a breast cancer does not eliminate or minimize the need of axillary lymph node staging. The status of the axillary lymph nodes influences the need and extent of lymph node surgery, the need for anticancer medications, and the need and extent of breast and axillary radiation. Consequently, the approach to lymph node management for cryoablation patients should generally parallel the lymph node management of patients treated with lumpectomy or mastectomy.

Like surgery patients, the options for lymph node management among cryoablation patients range from complete omission of lymph node surgery for women at low risk of axillary metastasis to sentinel node biopsy or axillary node dissection for women with a higher risk of lymph node metastasis or documented lymph node metastasis. Thus, women with low-risk, early-stage invasive breast cancer may be managed in accordance with the Society of Surgical Oncology’s Choosing Wisely recommendations, which advises omitting routine sentinel node biopsy in women aged 70 and older with stage I, hormone receptor positive, HER2/neu negative invasive breast cancer who have committed to taking anti-estrogen therapy [21]. Exceptions to this recommendation include women aged 70 and older with high grade tumors, high-risk recurrence scores, or low levels of estrogen and/or progesterone receptor expression for whom the risk of lymph node metastasis is higher and the benefits of anti-estrogen therapy lower.

Sentinel node biopsy should be routinely offered to younger clinically node negative women to detect or exclude the presence of microscopic lymph node metastasis. However, among these women, there exists the opportunity to use genomic testing to identify those with genomically low-risk cancers for whom lymph node monitoring with ultrasound may be considered in lieu of sentinel node biopsy. Conversely, women with normal appearing nodes but genomically high-risk tumors should continue to be advised to undergo sentinel node biopsy due to the elevated risk of microscopic lymph metastasis.

Patients with triple negative or HER2/neu positive invasive breast cancer and normal appearing lymph nodes should undergo sentinel node biopsy given the higher risk of microscopic lymph node metastasis and the implication of a positive node on the overall treatment plan. Among these patients, sentinel node biopsy should be performed after chemotherapy and/or anti-HER2/neu therapy if the patient received these anticancer medications prior to cryoablation. On the other hand, the cryoablation and sentinel node biopsy procedures may be performed before chemotherapy and/or anti-HER2/neu therapy if the patient is only willing to receive anticancer medications if the sentinel node biopsy is positive. Among patients who are resistant to receiving chemotherapy or anti- HER2/neu therapy, the finding of a positive sentinel node might motivate them to reconsider their decision to avoid systemic therapy.

**Figure 15:**
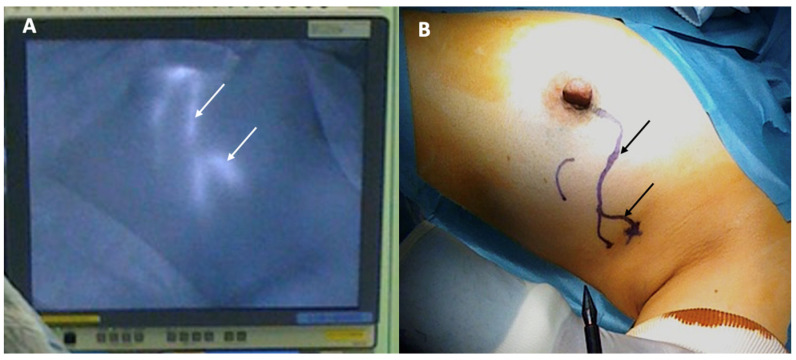
Photographs showing lymphatic mapping with a fluorescent tracer (**A**) and corresponding skin surface markings (**B**) identifying the predominant pattern of lymphatic drainage from the areola to the axilla. Arrows indicate the path of lymphatic channels.

Sentinel node biopsy is a lymph node staging procedure that aims to identify microscopic metastasis in individuals with clinically normal lymph nodes. Generally, one or two lymph nodes are excised and examined microscopically for breast cancer metastasis. Sentinel node biopsy may be performed using a radioactive substance and/or a blue dye tracer that is injected into the breast to identify the main channels through which lymphatic fluid (and potentially cancer cells) drains from the breast to the lymph nodes. Since the dominant pattern of lymphatic drainage of the breast typically courses just below the skin in the upper outer quadrant of each breast, the cryoablation provider must consider the implications of cryoablating a superficial upper outer quadrant cancer given the risk that ablation of tissue in the upper outer quadrant might disrupt lymphatic drainage of the radioactive and/or blue dye tracer to the axilla, which can interfere with sentinel node detection (Figure 15). Thus, when cryoablation and sentinel node biopsy are planned for such cancers, consideration should be given to either performing the sentinel node biopsy procedure before the cryoablation procedure or performing the sentinel node tracer injection prior to the cryoablation procedure followed within 24 h by the sentinel node biopsy procedure.

**Figure 16:**
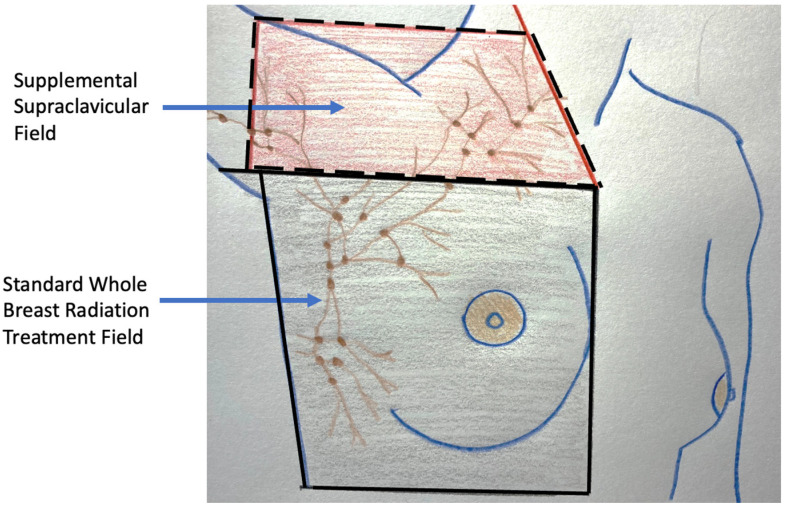
Figure showing radiation treatment field for standard whole breast (radiation area enclosed with solid lines) and the supplemental radiation field below and above the clavicle for a patient with multiple positive axillary lymph nodes (area enclosed with hash lines).

For patients with early-stage breast cancer and microscopically positive sentinel node nodes, the current standard of care after surgery is the addition of radiation to the lymph node region where as much as 30% of the residual lymph nodes may contain microscopic metastatic cancer (Figure 16) [22]. Without compromising cancer control, the use of radiation in this setting has significantly reduced the risk of chronic arm lymphedema (swelling) associated with surgical removal of the remaining axillary nodes. Although the use of axillary radiation instead of axillary node dissection has not been examined in breast cancer cryoablation clinical trials, substituting axillary radiation for complete lymph node removal reflects a growing trend in the field of breast oncology and is a reasonable consideration for selected cryoablation patients with microscopically positive sentinel nodes.

For patients with grossly positive axillary lymph nodes confirmed by needle biopsy, the current standard of care is either axillary lymph node dissection or upfront anticancer medications administered with the goal of downstaging or eradicating cancer from the affected lymph nodes to permit more limited lymph node surgery. Axillary lymph node dissection is one of the procedures most feared by breast cancer patients due to the risk of lifelong arm lymphedema. However, the following lymph node surgical procedures may be offered to lower the risk of arm lymphedema and to reduce the overall burden (e.g., acute and chronic pain) of lymph node surgery:

1. The extent of axillary surgery can be limited to the removal of positive lymph nodes detected preoperatively or intraoperatively with the goal of leaving intact all normal-appearing nodes to minimize the burden of extensive lymph node surgery [23]. In this scenario, the addition of radiation therapy to the remaining lymph axillary nodes can manage the risk of lymph node recurrence while also reducing the risk of arm lymphedema.
2. Lymph node surgery can be performed under local anesthesia with sedation to minimize anesthesia side effects and expedite recovery from surgery. The procedure can be combined with pectoralis nerve blocks to reduce intraoperative and post-operative pain.
3. Axillary reverse mapping can be performed by surgeons to facilitate visualization and avoidance of the arm lymphatic drainage at the time of lymph node surgery. Axillary reverse mapping typically involves the injection of blue dye into the arm near the axilla. Absorption of the blue dye by the arm lymphatics causes the lymphatic vessels and lymph nodes draining the arm to turn blue. Blue-stained lymphatic vessels and lymph nodes detected during surgery may be avoided and preserved if they are clinically normal and not identified as a sentinel node. Axillary reverse mapping has been shown to significantly reduce the risk of lymphedema in patients undergoing lymph node surgery, especially when removal of multiple axillary lymph nodes is required [24,25].

**Figure 17:**
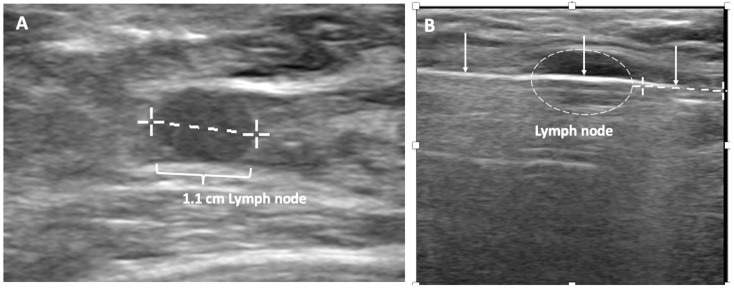
(**A**) shows a grossly abnormal 1.1 cm axillary lymph node prior to insertion of cryoprobe. (**B**) shows an abnormal lymph node (outlined with hash marks),the cryoprobe traversing the lymph node (white line indicated by arrows), and projection of the cryoprobe tip beyond the distal end of the lymph node (linear hash marks) prior to beginning first freeze cycle.

Despite the surgical advancements to minimize the burden of lymph node surgery, some patients with positive lymph nodes continue to refuse lymph node surgery. In the context of patient centered care, some patients with limited nodal disease may be considered for lymph node cryoablation in lieu of surgery (Figure 17). Lymph node cryoablation entails several important considerations. Unlike cancers in the breast, the encapsulated nature of lymph nodes obviates the need for cryoablation of a wide surgical margin. Thus, cryoablation freeze times may be cut short when the dimensions of the iceball extend 5 mm beyond the lateral edges of the lymph node. However, lymph node cryoablation is not without hazard. Although lymph node cryoablation is a minimally invasive procedure, the elongated shape of the oval iceball typically causes the cryoablation zone of necrosis to extend 10–15 mm beyond the near and far edges of the lymph node, which can inadvertently cause cryoablation or injury to adjacent nodes, vessels, or nerves. Furthermore, since cryoablation does not permit axillary reverse mapping, lymph node cryoablation is comparatively less targeted and potentially more morbid than selectively removal of grossly abnormal axillary lymph nodes.

Additionally, although lymph node cryoablation might effectively manage individual low-lying axillary nodes, cryoablation of multiple axillary nodes or cryoablation of lymph nodes in the upper axilla can increase the risk of lymphedema due to collateral disruption of arm draining lymphatic structures. Furthermore, attempted ablation of nodes near the large vessels in the upper axilla has a greater risk of incomplete ablation due to the high volume of warm blood flow through the axillary artery and veins that prevents the iceball from reaching an extremely cold temperature. Lastly, the presence of a grossly abnormal lymph node suggests that nearby normal-appearing nodes might be microscopically positive. Consequently, these patients would benefit from radiation therapy to reduce the risk of recurrence in the breast and lymph nodes.

Anticancer medications or systemic therapy. Unlike surgery and cryoablation which aim to minimize the risk of recurrence within the breast and axilla nodes, systemic therapy is given for invasive breast cancer with the goal of improving overall survival by preventing growth of microscopic metastatic cancer deposits, which might already exist in the lung, liver, bone, brain, and other organs at the time of the initial cancer diagnosis. The probability of distant metastasis increases with higher cancer stage and more aggressive tumor biology. In general, distant metastases originate from the primary breast cancer long before the breast cancer diagnosis is made. Their microscopic size makes them virtually undetectable at the time of diagnosis, which explains why CT scans, PET/CT scans, and bone scans are generally discouraged for women with early-stage breast cancer. However, in the absence of effective therapy, these distant metastases, if present, tend to grow to a larger size and become detectable within the first 3 years after the original diagnosis.

Given the importance of systemic therapy for improving breast cancer survival, it is imperative for cryoablation providers to educate patients about the risks of distant metastasis and recurrence and the potential benefits of systemic therapy. This includes a high-level discussion of the potential benefit of anti-estrogen therapy for hormone-receptor positive breast cancers, chemotherapy for clinically high-risk or genomically high-risk invasive breast cancers, anti-HER2/neu therapy for HER2/neu-positive invasive breast cancers, immunotherapy for PD-1-positive triple negative invasive breast cancers, and PARP inhibitors and platinum agents for gene mutation-positive invasive breast cancers. At a minimum, patients should be offered a referral to a medical oncologist for consideration of one or more of these treatment regimens. If genomic testing has yet to be performed for a patient with hormone receptor positive, HER2/neu negative invasive breast cancer, it would behoove the cryoablation provider to confirm that adequate tissue is available from the original diagnostic needle biopsy to preserve the option of genomic testing. If necessary, additional needle biopsies of the cancer site may be performed at the time of cryoablation to obtain additional tissue for genomic testing.

Many cryoablation patients are open to receiving anti-estrogen therapy, anti-HER2/neu therapy, immunotherapy, and chemotherapy when they have been provided a realistic estimation of the risk of cancer recurrence. For more reluctant patients, shared decision-making may lead the provider and patient to consider modifications of standard systemic therapy regimens in favor of agents that may be more compatible with the patient’s level of risk-tolerance. Potential options include omission or substitution of specific medications, treatment of genomically high-risk cancer with anti-estrogen therapy alone without chemotherapy, or treatment of HER2/neu positive invasive breast cancer with anti-HER2/neu antibodies without chemotherapy.

Historically, breast cancer was routinely managed with upfront surgery followed by adjuvant systemic therapy. In the 1980′s, studies examining the sequencing of surgery and chemotherapy found that upfront or neoadjuvant chemotherapy often made the cancer smaller and more easily managed with lumpectomy without altering overall survival prospects. Thus, upfront chemotherapy was established as a strategy for expanding the opportunity for breast preservation in women who would otherwise require mastectomy due to large tumor size.

With regards to triple negative and HER2/neu positive invasive breast cancer, optimal sequencing of cancer therapy has changed considerably since the 1980′s. Recent clinical trials using more effective anticancer mediations demonstrate the potential for marked improvement in overall survival with upfront chemotherapy and/or anti-HER2/neu therapy [26,27]. In fact, a large proportion of these patients experience a pathologic complete response, meaning that they are found to have no detectable disease in the breast or lymph node specimens after completing neoadjuvant systemic therapy, which suggests that microscopic disease in distant organs is also likely to have been eradicated [28,29]. For both HER2/neu positive and triple negative breast cancer, a pathological complete response is a strong indicator of improved breast cancer specific and overall survival [27]. Given the potential of improved survival with upfront chemotherapy, it is imperative that women with HER2/neu positive breast cancer (>2 cm), triple negative invasive breast cancer (>1 cm), or node positive breast cancer, should be referred for anti-HER2/neu therapy and/or chemotherapy prior to undergoing cryoablation or surgery.

An additional reason for recommending neoadjuvant systemic therapy for triple negative and HER2/neu positive invasive breast cancer is that individuals who are found to have residual disease after completing neoadjuvant systemic therapy may achieve a survival benefit from supplemental systemic therapy after surgery. Initial surgical removal or cryoablation of triple negative and HER2/neu positive invasive breast cancer eliminates the possibility of assessing the cancer’s response to neoadjuvant systemic therapy, which makes it impossible to identify patients that might benefit from an expanded systemic therapy regimen.

**Figure 18:**
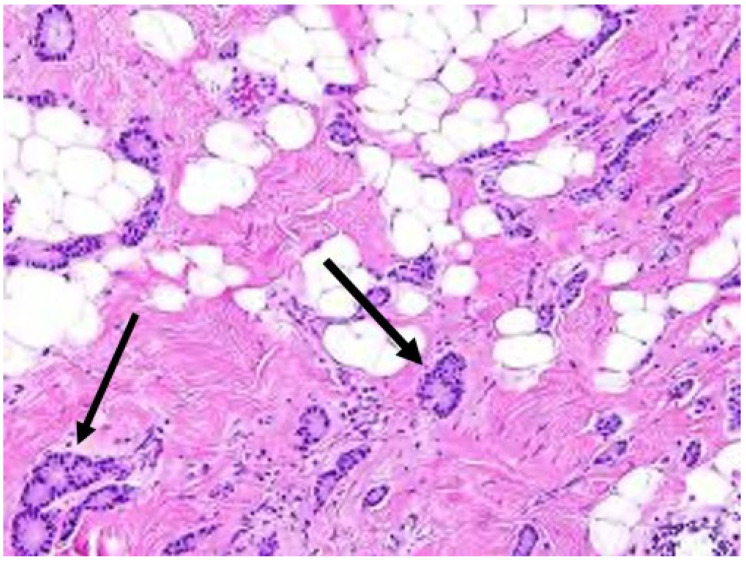
Arrows indicate locations of residual cancer cells persisting after completion of upfront chemotherapy.

**Figure 19:**
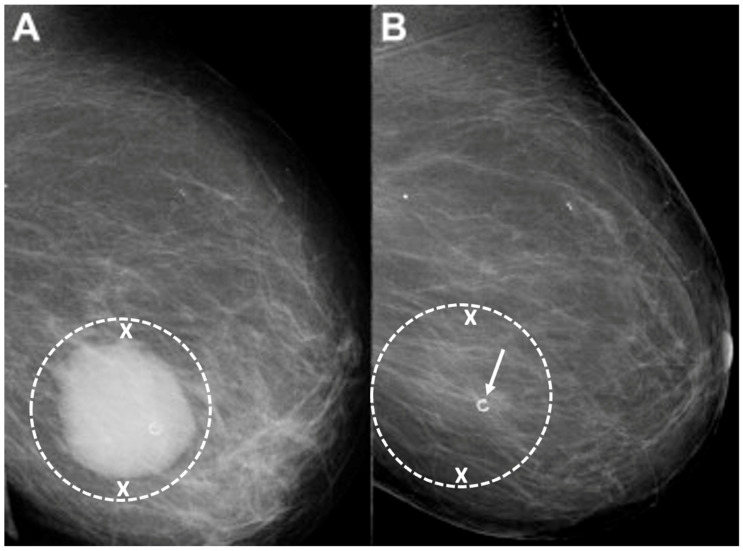
Mammograms performed before (**A**) and after (**B**) chemotherapy showing representative locations (indicated by “X” marks) where bracketing biopsy site markers can be placed before starting chemotherapy to mark the edges of the white, dominant cancer dimensions in the event the mass is no longer visible after chemotherapy. Figure B shows the same breast after completing chemotherapy showing complete resolution of the mass and the representative locations of bracketing site markers (indicated by “X” marks) placed before chemotherapy to outline the original tumor dimensions.

Patients treated with neoadjuvant systemic therapy have traditionally been excluded from cryoablation clinic trials in large part due to concerns that systemic therapy could compromise the efficacy of cryoablation by making the cancer more difficult to target. However, selected patients treated with neoadjuvant systemic therapy may preserve the option of cryoablation if the following precautions are undertaken:

1. Anticipate disappearance of ultrasound-visible markers. Most ultrasound-visible biopsy site markers have a “permanent,” non-absorbable metal component and an absorbable (collagen or hydrogel) component that confers ultrasound-visibility. However, ultrasound visibility diminishes over time and is significantly reduced after 12 weeks [30]. Consequently, the most practical approach for patients likely to be managed with neoadjuvant systemic therapy is to place standard, non-ultrasound-visible biopsy site markers at the time of the original diagnostic needle biopsy followed later by the adjacent placement of ultrasound-visible biopsy site markers in the weeks immediately preceding the cryoablation procedure.
2. Repeat needle biopsy of cancer site prior to cryoablation. Following neoadjuvant systemic therapy, detection of residual cancer cells at the original cancer site can profoundly impact the patient’s subsequent management. Consequently, the cryoablation provider should seek the patient’s consent to obtain one or more needle biopsies of the original tumor site to determine the presence or absence of residual living cancer cells. Even patients with no radiographically visible cancer after neoadjuvant systemic therapy might be found on needle biopsy to have residual microscopic disease, the detection of which might influence their subsequent management (Figure 18).

3. Target cryoablation of the original tumor volume. Although neoadjuvant systemic therapy might induce the complete radiographic disappearance of the cancer, individual cancer cells or microscopic clusters of cancer cells could persist throughout the span of the original cancer (Figure 19). Therefore, targeting the original tumor volume plus a surrounding ablation margin has a greater potential to achieve complete ablation at the cancer site. This strategy contrasts with how patients are managed surgically after neoadjuvant systemic therapy, where the ability to perform microscopic examination of the surgical margins permits the initial excision of a smaller volume of tissue followed later by margin re-excision if the microscopic assessment reveals residual cancer at the surgical margin.

The Abscopal Effect. Many women are drawn to cryoablation with the hope that breast cancer cryoablation will induce an anticancer immune response to breast cancer cells located at distant sites. Conceptually, an abscopal effect occurs when natural immune cells arriving to clean up a cryoablated cancer induce an anti-cancer immune response that prevents growth or causes regression of immunologically identical cancer cells that had metastasized to other parts of the body.

Preliminary studies in human breast cancer suggest that cryoablation of distant metastatic sites improves survival in women with Stage IV breast cancer which might partially be attributable to an abscopal effect. Currently, the standard of care for Stage IV breast cancer discourages routine surgical removal or ablation of distant metastatic sites based on the view that these interventions do little to halt the overall progression of clinically occult systemic disease. However, this principle has been challenged by a retrospective study by Nui et al. who reported improved overall survival among 121 women with stage IV breast cancer treated with cryoablation of one or more metastatic sites [31]. Significantly longer overall survival was observed among women treated with cryoablation compared to chemotherapy alone (27 months). Among women receiving cryoablation, significantly longer overall survival was observed in those treated with cryo-immunotherapy (83 months) and cryotherapy alone (55 months) compared to cryoablation combined with chemotherapy (43 months). Among patients with multiple metastatic sites, overall survival was also significantly longer for patients who underwent cryoablation of multiple metastatic sites (76 months) compared to those who received cryoablation of only one of the metastatic sites (48 months). Although the study may be criticized for its non-randomized nature and the lack of published pathology data, extended overall survival amongst a cohort of women expected to have relatively short long-term survival strongly suggests that cryoablation may be beneficial in controlling systemic disease and that overall survival can be enhanced by the synergistic effects of immunotherapy. 

Ongoing research in humans strongly suggests that the best predictors of a potential abscopal response is the presence of an abundance of natural immune cells, including T cells, in the vicinity of a cancer [32]. Another important predictor is T-cell expression of PD-1 checkpoint proteins, which are now known to be important regulators of the immune response. A pilot study combining cryoablation and a single dose of ipilimumab, a checkpoint inhibitor, demonstrated a modest but favorable anti-cancer systemic immune response [33]. Building on this initial experience, the synergistic effect of cryoablation and checkpoint inhibitors is currently being explored by a prospective phase II clinical trial evaluating perioperative use of not just one but two checkpoint inhibitors (ipilimumab and nivolumab combined) in patients with triple negative invasive breast cancer that have persistent radiographically detectable disease after completing neoadjuvant chemotherapy [34]. The study calls for all subjects to undergo cryoablation of the residual breast cancer followed by a 5-day course of ipilimumab and nivolumab. A subsequent core needle biopsy of the cryoablation site is followed 7–10 days later by lumpectomy or mastectomy. After surgery, participants receive nivolumab every 2 weeks for 3 additional doses. All lumpectomy and mastectomy specimens will be evaluated to measure the local immune response. Patients will also be monitored for 3 years to determine their breast cancer survival and overall survival. Another ongoing, phase I, study in locally advanced and stage IV triple negative breast cancer aims to evaluate the safety, feasibility, and long-term outcome of cryoablation of the primary breast tumor followed by atezolizumab, a PD-L1 inhibitor, and nab-paclitaxel, a chemotherapy agent [35].

Much remains to be learned about how to best optimize the interaction between cryoablation and immunotherapy in humans to promote a clinically meaningful local and systemic immune response. However, given the lack of randomized or case-controlled data supporting cryo-immunology in humans, patients should be wary of cryoablation providers who offer cryoablation with “immunotherapy” outside of a formal IRB-approved clinical trial. 

Radiation therapy. Local recurrence in the breast is a well-recognized risk of breast preserving procedures. Consequently, radiation therapy is generally recommended after lumpectomy to reduce the risk of recurrence arising from microscopic cancer cells that may persist in the perimeter of the cancer site despite achieving negative margins. For example, in the landmark study, NSABP-B06, women randomized to lumpectomy alone experienced a 10-year local recurrence rate of 29.2% compared to 10.0% for women treated with lumpectomy plus whole breast radiation therapy—a 66% relative risk reduction with radiation therapy [36]. The benefit of radiation therapy was even greater among women with node-positive disease, revealing a 13% 15-year local recurrence rate for lumpectomy plus radiation therapy versus a 46% 15-year local recurrence rate for lumpectomy without radiation therapy. Although recent advances in the management of breast cancer have reduced 10-year and 15-year local recurrence rates, contemporary studies continue to demonstrate a 60–70% relative reduction in local recurrence rates when radiation therapy is added to lumpectomy.

For lumpectomy patients, radiation therapy typically consists of a 4–6-week course of daily radiation therapy doses, or fractions, administered to the entire breast. Although the logistics and efficacy of breast radiation therapy have not been directly examined in cryoablation clinical trials; it is reasonable to conclude that the general principles guiding whole breast radiation therapy after lumpectomy would also apply to women treated with breast cryoablation.

Internationally, the vast majority of cryoablation clinical trial participants treated with radiation therapy after cryoablation have received the standard 6-week course of whole breast radiation therapy with no significant adverse events. Global experience with the 4-week course of hypofractionated, whole breast radiation therapy is gradually increasing and will likely provide further insights when employed in upcoming cryoablation clinical trials. Presently, there are no published reports of external beam based accelerated partial breast radiation in patients treated with breast cryoablation. The lack of an open surgical cavity following cryoablation excludes the use of intraoperative radiation therapy and other forms of intracavitary partial breast radiation therapy.

Lumpectomy patients treated with whole breast radiation therapy are sometimes administered an additional targeted dose of radiation to the surgical margins (called a “boost”) with the goal of further reducing the risk of recurrence. Medical evidence strongly supports the use of a radiation boost after lumpectomy for women ≤ 50 years of age, women aged 51–70 years with high-grade invasive breast cancers, and in the setting of positive margins [37]. It is reasonable to conclude that the same principles would apply to patients treated with breast cryoablation. However, the inability to assess surgical margins following cryoablation should not automatically lead to the conclusion that the ablation margins are positive and should therefore be treated with a radiation boost.

Ultimately, the magnitude of benefit of radiation therapy with or without a boost depends on the estimated risk of local recurrence after lumpectomy. As such, there are populations of women for whom radiation therapy can be safely omitted. For example, a randomized controlled trial demonstrates minimal radiation therapy benefit after lumpectomy in women 70 years and older with stage I, grade 1 or 2, ER+ and/or PR+ positive, HER2/neu negative invasive breast cancer who received anti-estrogen therapy [38]. Similarly, the LUMINA trial found minimal radiation benefit after lumpectomy in women 60 years and older with stage I, ER+ and/or PR+ positive, HER2/neu negative, low risk (Luminal A) invasive breast cancer treated with anti-estrogen therapy [39]. In these patient populations, one may reasonably conclude that radiation therapy would have minimal impact on local control among cryoablation patients who have committed to taking anti-estrogen therapy. Nonetheless, it is impossible for patients to make an informed decision about radiation therapy if they are unaware of the pros and cons of omitting it. Therefore, accountable providers have the responsibility to communicate to patients their estimated risk of recurrence with and without radiation therapy based on the best data available.

For women with invasive breast cancer, there are currently no validated commercially available genomic assays for estimating the rate of local recurrence after lumpectomy or cryoablation. However, the 10-year risk of local recurrence among women with early-stage invasive breast cancer can be estimated using the online tool, IBTR! 2.0 (https://www.evidencio.com/models/show/1386, accessed on 12 August 2023), which integrates seven different variables (age, tumor size up to 10 cm, tumor grade, margin status, the presence of lymphovascular invasion, use of chemotherapy, and use of anti-estrogen therapy) to estimate the relative benefit of radiation therapy and anti-estrogen therapy [40]. A key shortcoming of the tool is its tendency to overestimate the risk of local recurrence, a limitation that is perhaps less harmful than underestimating the risk [41]. Additional shortcomings include its lack of utility for women with triple negative, HER2/neu positive, or lymph node-positive breast cancer. Notwithstanding these limitations, the online calculator does provide a means of assessing local recurrence rates for the majority of women diagnosed each year with early-stage invasive breast cancer, particularly those who are likely candidates for cryoablation.

Patients with DCIS may benefit from two commercially available genomic assays, DCISionRT and OncotypeDX, for estimating the 10-year risk of invasive and DCIS recurrence with and without radiotherapy. DCISionRT is validated for women ≥ 30 years of age with DCIS measuring ≤ 7 cm. OncotypeDX DCIS is validated for women ≥ 18 years of age with DCIS measuring ≤ 2.5 cm. Although these tests were not validated in patients treated with cryoablation, one can reasonably infer that these genomic assays would be applicable for estimating the risk of recurrence following breast cryoablation.

Follow-up. There is no standard post-treatment follow-up protocol for breast cancer survivors treated with cryoablation. However, in keeping with the standard protocol for women treated with lumpectomy, women treated with cryoablation would be expected to undergo annual clinical breast examinations and annual mammograms. Annual mammograms may be supplemented with diagnostic ultrasound or contrast-enhanced breast MRI depending on the clinical findings, breast density, risk factors, and features of the original cancer (e.g., calcifications, mammographically occult cancer, MRI enhancement). Some patients may refuse mammography and contrast-enhanced breast MRI due to concerns about radiation or gadolinium exposure. For such patients, a customized post-cryoablation imaging regimen might be required. Regardless of the regimen, the purpose of follow-up imaging is to detect residual or recurrent breast cancer which, if present, is most likely to be detected in the cryoablation zone of inflammation surrounding the cryoablation zone of necrosis, a likely result of interval progression of pre-existing multifocal disease that was imaging-occult at the time of the original cryoablation procedure. 

A major challenge remaining with the imaging follow-up of a cryoablation patient is the relative lack of experience amongst radiologists in interpreting post-cryoablation imaging studies. In fact, the current level of experience mirrors the learning curve that many radiologists endured in the early days of lumpectomy and radiation therapy. As expressed in a representative medical journal article, interpretation of mammograms after lumpectomy “requires knowledge of the mammographic patterns of breast cancer and scar formation, an appreciation of the alterations that occur in the mammogram after breast irradiation, and an understanding of the benign changes that can mimic new breast cancer in these women [42]”. These remarks reflect current sentiments about women treated with breast cryoablation.

Where radiology cryoablation experience is lacking, patients may need to travel to breast imaging centers with greater expertise in interpreting post-cryoablation imaging studies, particularly the initial set of post-cryoablation studies which are the most challenging to interpret. Once an “expert” reviews and endorses a new post-cryoablation imaging baseline, other radiologists will find it easier to read subsequent imaging studies. 

**Figure 20:**
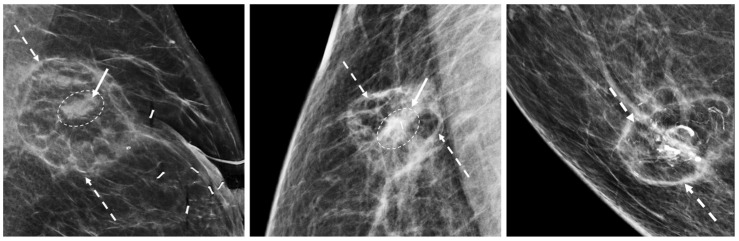
Post-cryoablation mammograms showing thin walled, spherical scars or cryoballs (broken arrows) and centrally located tumor ghosts (indicated by solid arrows and encircled with hash marks).

**Figure 21:**
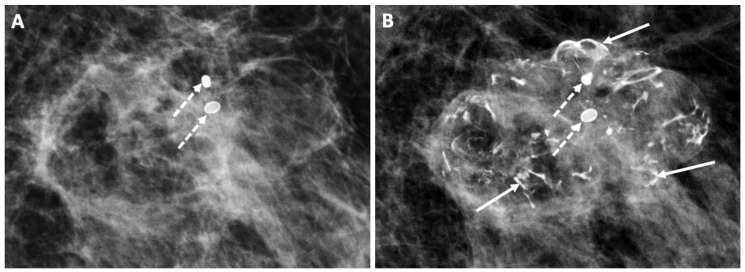
Follow-up mammograms showing an example of increasing macrocalcifications indicative of fat necrosis developing 1 year (**A**) and 2 years (**B**) after cryoablation. Only two macrocalcifications were present in 1 year (broken arrows), but by year 2, numerous additional macrocalcifications had developed (solid arrows).

The mammographic appearance of the cryoablation site is typically characterized by a thin, spherical scar or cryoball that roughly corresponds to the dimensions of the iceball that was achieved at the time of cryoablation or to the dimensions of the cryoablation zone of inflammation that was ultimately achieved. The “eggshell” appearance of the cryoball is more apparent in fatty breasts but more subtle in dense breasts (Figure 20) [43]. Another common post-cryoablation mammographic feature is the persistence of a tumor “ghost,” which is the visible remnant of the necrotic cancer mass that gradually resorbs over time. Although the tumor ghost is expected to gradually disappear, microcalcifications associated with the malignancy are expected to persist even when the surrounding cancer cells have been completely resorbed. Microcalcifications are essentially fossilized remnants of dead cancer cells that have become calcified and petrified, and as such are not digestible by the immune cells. However, it is possible for new microcalcifications to appear. Most often, new microcalcifications or macrocalcifications within or adjacent to the cryoablation site indicate the development of benign fat necrosis (Figure 21), a common manifestation of tissue trauma. It is also possible that new microcalcifications within or adjacent to the cryoball could indicate the presence of recurrent disease. In either case, a stereotactic or mammographic guided needle biopsy might be needed to distinguish between recurrence and fat necrosis.

**Figure 22:**
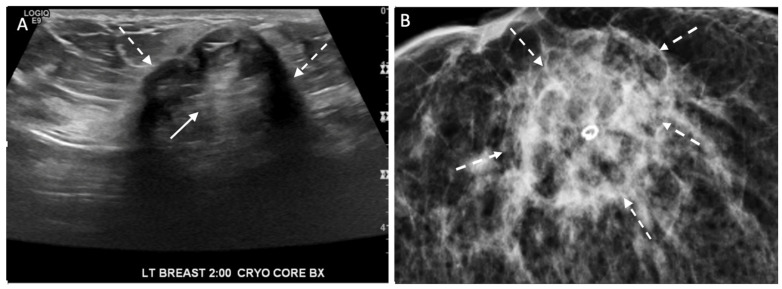
Ultrasound (**A**) and corresponding mammogram (**B**) of cryoablation site performed 6 months after cryoablation with broken arrows indicating outer edges of the cryoball and solid arrow (**A**) at site of central tumor ghost.

Ultrasound is commonly used to monitor the cryoablation site, but the presence of a tumor ghost and the significant tissue distortion caused by the cryoablation procedure make it particularly challenging for ultrasound to exclude the presence of residual or recurrent cancer within the cryoball (Figure 22). However, in contrast with mammography, ultrasound of the cryoablation site consistently demonstrates a distinct, hyperechogenic border corresponding to the edges of the cryoball, regardless of breast density. Ultrasound can also detect new hypoechoic masses at the perimeter of the cryoball that might indicate tumor recurrence.

**Figure 23:**
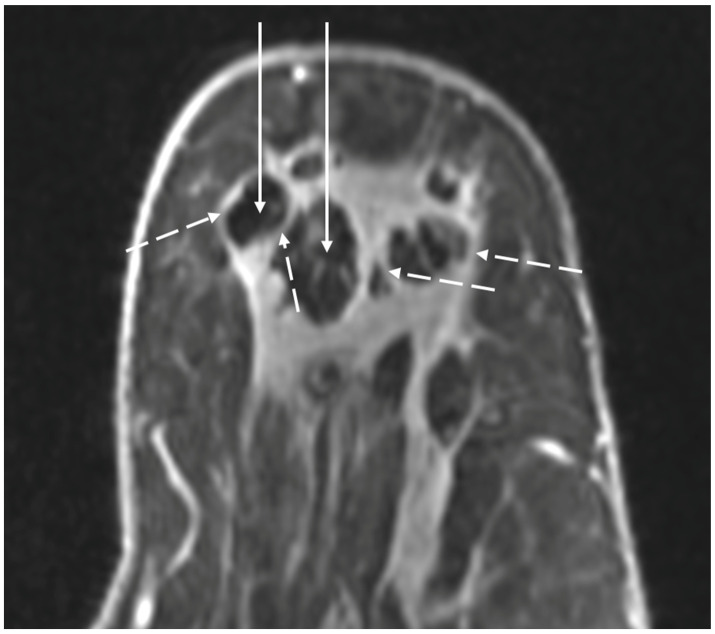
MRI images showing spherical rim enhancement (broken arrows) surrounding relatively avascular cryoablation zones of necrosis (solid arrows).

Contrast-enhanced breast MRI is generally the most useful imaging study for assessing the extent of the cryoablation zone of injury and for detecting the presence of residual or recurrent disease. By MRI, the outer edge of the cryoball typically appears as a thin, relatively uniform, spherical rim of enhancement surrounded by a relatively avascular cryoablation zone of necrosis containing the centrally located tumor ghost (Figure 23) [44,45,46,47,48]. If multiple overlapping cryoablations are performed, the overlapping cryoballs will produce a more highly variable pattern of rim enhancement depending on the orientation of the cryoablation zones.

Contrast-enhanced MRI could also reveal persistent blood flow within the tumor ghost, which might suggest the presence of residual disease. The degree of MRI enhancement (i.e., the degree of blood flow) in the tumor ghost is partly dependent on the pattern of cancer enhancement pre-cryoablation as well as the time interval between the cryoablation and the follow-up breast MRI. Contrast-enhanced breast MRI performed in the first two months after cryoablation generally demonstrates a subtle, non-specific pattern of blood flow within the tumor ghost, characteristic of healing wounds, which makes it difficult to exclude the presence of microscopic cancer [49]. However, by 6–24 months after cryoablation, contrast-enhanced breast MRI typically shows complete loss of enhancement and the absence of a tumor ghost, either of which strongly suggests the absence of residual or recurrent cancer. In a small study by Kawamoto et al., the diameter of the cryoablation zone of necrosis was observed to have decreased by 58% over 2 years. Adjacent muscles typically exhibit various degrees of inflammation indicative of a “thermal burn,” which typically resolves over 6 months [50].

Depending on the clinical stage, FDG-PET/CT might sometimes be performed to exclude the presence of distant disease. Although FDG-PET/CT is expected to have poor resolution of breast anatomy, fat necrosis and inflammation at the cryoablation can produce a low level of activity on FDG-PET/CT that might be difficult to distinguish from recurrence or residual disease. Among patients undergoing FDG-PET/CT within 12 months of cryoablation, Adachi et al. report a median maximum standard value unit (SUVmax) of 1.36, which is generally considered benign. In general, the SUVmax was typically significantly higher in younger women, pre-menopausal women, and in those with higher breast density [51].

**Figure 24:**
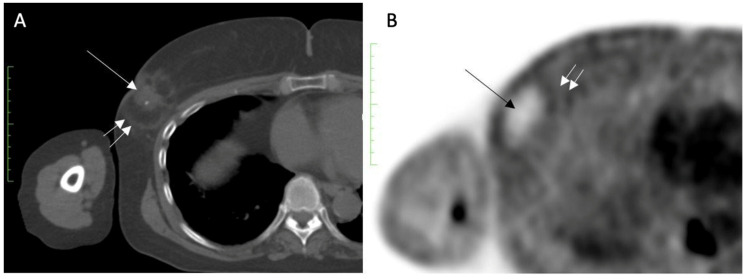
(**A**) shows an FDG-PET/CT performed 1 month after cryoablation of a 3 cm ER+, PR+, HER2/neu negative invasive ductal carcinoma, with a central tumor ghost (single arrow) and edge of spherical cryoball (double arrow). (**B**) shows a significant reduction in FDG-PET/CT at the cryoablation site (single arrow) compared to background levels of activity in the normal surrounding tissues (double arrow).

Despite its inability to completely exclude recurrence or residual disease, serial FDG-PET/CT performed for other reasons might demonstrate a loss or decrease in activity at the cryoablation site, which is considered a benign feature. For example, Figure 24A shows FDG-PET/CT images performed 1 month after cryoablation of a 3 cm ER+, PR+, HER2/neu negative invasive ductal carcinoma, revealing a significant loss in FDG- PET/CT at the cryoablation site compared to background levels of activity in the normal surrounding tissues (Figure 24B).

At present, there are no publications in the breast oncology literature that establish the sensitivity of mammography, ultrasound, and/or MRI in detecting residual disease following cryoablation. However, data from the liver cancer literature found MRI and CT combined to be only 30–60% effective at detecting residual cancer after ablation among the subset of women with confirmed residual cancer. There are no data suggesting that breast imaging would perform as poorly as liver imaging post-cryoablation. However, the persistence of residual findings (e.g., tumor ghost, MRI enhancement, and FDG-PET/CT-activity) at the cryoablation site makes it difficult for imaging alone to exclude residual disease, which sometimes makes it necessary to perform a needle biopsy of the cryoablation site to definitively exclude residual or recurrence cancer.

There is no widely accepted policy regarding performance of a routine needle biopsy of the cryoablation site as part of the post-cryoablation follow-up protocol. However, there are several potential advantages of routine needle biopsies. For all parties, a benign core needle biopsy of the cryoablation site confirms the absence of residual or recurrent cancer. For patients and providers, a benign core needle biopsy result allays concerns that might remain regarding the dominant breast lump or “cryoball” that naturally forms as a result of cryoablation, and which may persist for multiple years following the cryoablation procedures. For medical oncologists and radiation oncologists, a benign needle biopsy result confirms the absence of residual disease before continuing with adjuvant systemic therapy and/or radiation therapy. For many inexperienced radiologists, the presence of a persistent and often enlarged mass at the cryoablation site leads them to assign a BI-RADS score of 6 to subsequent imaging studies, which signifies the presence of a known cancer for which treatment is still needed. This can cause confusion among oncologists and anxiety among patients. Therefore, if the radiologist is uncertain about downgrading a post-cryoablation study to BI-RADS 2 or 3, indicating a benign finding for which follow-up imaging is recommended, then the performance of a routine post-cryoablation needle biopsy might be needed until the radiologist has acquired sufficient experience to downgrade post-cryoablation studies based on imaging appearance alone. 

**Figure 25:**
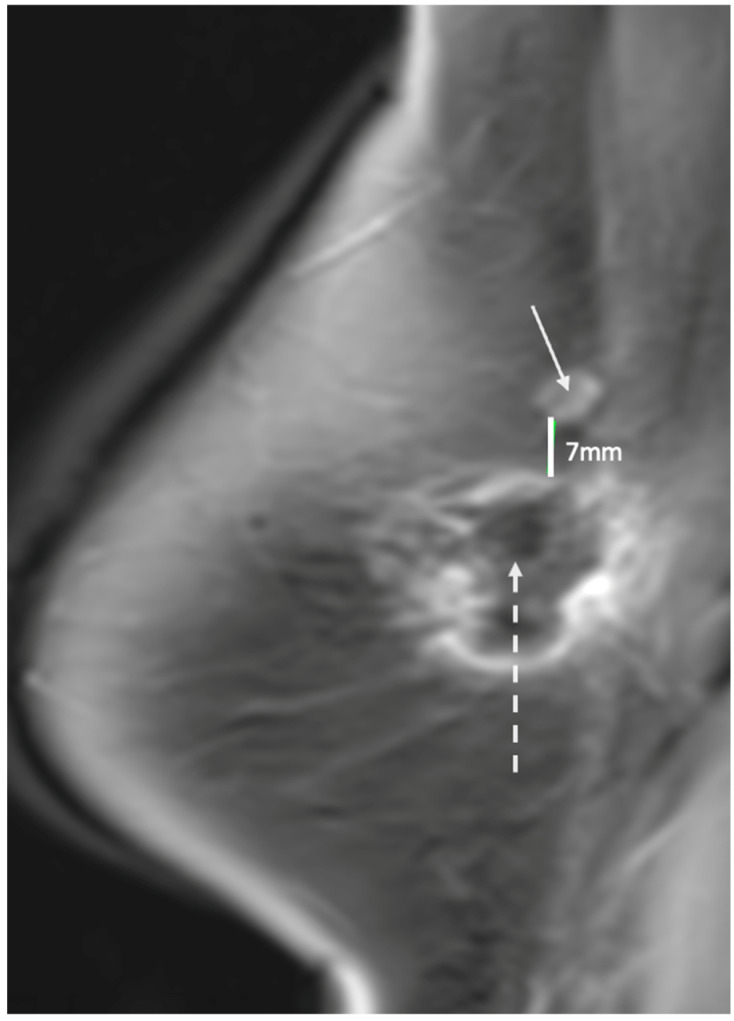
Contrast enhanced breast MRI showing new area of suspicious enhancement (indicated by solid arrow) suggestive of a recurrence 7 mm superior to spherical cryoablation zone of necrosis (indicated by broken arrow).

Management of local recurrence. It is a reality of breast oncology that a subset of patients treated with breast conserving procedures will experience a recurrence of cancer in the breast. The potential of recurrence within the breast is particularly relevant for patients with high-risk disease. Similar to the risk of distant recurrence, recurrences within the breast or axilla are more likely to develop during the first 3 years after cryoablation. In most instances, local recurrences will arise in the perimeter of the cryoablated cancer, within or just beyond the cryoablation zone of inflammation in tissue that appeared normal at the time of cryoablation, but which, in retrospect, contained microscopic disease (Figure 25). Local recurrences leave the patient and her provider with new healthcare decisions regarding the management of the recurrence. Fortunately, patients that experience a local recurrence after cryoablation generally retain all treatment options that were available at the time of the original diagnosis. Depending on the extent of recurrence, the patient may undergo mastectomy, lumpectomy, or repeat cryoablation targeting the recurrence. If the patient had previously declined anti-estrogen therapy and/or radiation therapy, perhaps she would perhaps be more open to considering anti-estrogen therapy and/or radiation therapy for the management of the recurrence.

## 4. Conclusions

Cryoablation is emerging as a minimally invasive alternative to breast conserving surgery for the management of breast cancer. Despite ongoing clinical trials and increasing acceptance of cryoablation in the medical community, there are no established guidelines or consensus statements informing the optimal multidisciplinary management of the breast cancer cryoablation patient. The lack of guidance contributes to confusion regarding the optimal diagnostic work-up, pre-cryoablation assessment, axillary management, and post-cryoablation follow-up, as well as the optimal use and timing of systemic therapy and radiation therapy. This overview aims to fill the knowledge void by presenting an evidence-based discussion of the topic that can inform physicians and patients today and provide an outline for a future breast cancer cryoablation practice guideline. Furthermore, given the potential for disparate care amongst different cryoablation providers, this discussion aims to advance uniform and technically skilled, multidisciplinary management of the breast cancer cryoablation patient, regardless of the treatment provider.

## Figures and Tables

**Table 1:** Pragmatic applications of breast cancer cryoablation.

		Cryoablation as Definitive Therapy	Cryoablation as Stopgap Therapy
A.	Stage I, clinically node-negative invasive breast cancer	Consider	Possibly Consider
B.	Stage I & II, clinically node-negative invasive breast cancer	Consider	Consider
C.	Stage III, clinically node-negative or node-positive invasive breast cancer	Possibly Consider	Consider
D.	Stage 0, ductal carcinoma in situ	Consider	Consider
E.	Locally recurrent invasive breast cancer or ductal carcinoma in situ	Consider	Consider
F.	Management of the breast primary in Stage IV breast cancer	Consider	Consider

**Table 2:** Checklist of key quality metrics related to the diagnostic work-up, pre-treatment planning, and follow-up of breast cancer cryoablation patients.

**Initial Diagnostic Work-up**
□ Mammograms to assess extent, including the presence or extent of suspicious microcalcifications □ Ultrasound of breast to assess extent and ultrasound visibility of cancer □ Ultrasound of axilla to detect suspicious axillary nodes □ Consideration of contrast-enhanced breast MRI to assess extent of disease □ Needle biopsy of suspicious breast mass(es) or calcifications □ Needle biopsy of a suspicious axillary node □ Biopsy site marker placement at needle biopsy sites □ Biomarker testing performed in needle biopsy specimen
**Pre-Treatment Planning**
□ Accountable provider(s) identified □ Review of biomarkers to determine optimal treatment sequence □ Assessment of risk of recurrence within the breast □ Consideration of genomic testing of invasive breast cancer to assess risk of distant metastasis and potential benefit of chemotherapy □ Consideration of genomic testing of ductal carcinoma in situ for assessment of risk of local recurrence □ Genetic counseling and testing based on NCCN guideline □ Staging for distant metastasis based on NCCN guideline □ Assessment of eligibility for cryoablation clinical trials □ Pre-cryoablation placement of ultrasound-visible biopsy site marker(s) if portions of cancer are not ultrasound-visible □ Assessment of mammograms for “clip migration” □ Consideration of placement of additional biopsy site markers prior to pre-cryoablation anti-cancer medications □ Consideration of lymph node monitoring without surgery for non-high-risk breast cancer □ Consideration of sentinel node biopsy for moderate to high-risk breast cancer □ Consideration for excision vs. ablation of suspicious or positive axillary nodes □ Referral to medical oncology for consideration of anti-cancer medications before or after cryoablation
**Post-Treatment Follow-up**
□ Referral to radiation oncology for consideration of radiation therapy □ Referral to or follow-up with medical oncology for consideration of systemic therapy □ Establishment of a plan for follow-up imaging □ Consideration of post-cryoablation needle biopsy to confirm the absence presence of residual disease

## Data Availability

Not applicable.
